# Individuality, phenotypic differentiation, dormancy and ‘persistence’ in culturable bacterial systems: commonalities shared by environmental, laboratory, and clinical microbiology

**DOI:** 10.12688/f1000research.6709.2

**Published:** 2015-09-07

**Authors:** Douglas Kell, Marnie Potgieter, Etheresia Pretorius

**Affiliations:** 1 School of Chemistry and The Manchester Institute of Biotechnology, The University of Manchester, Manchester, Lancashire, M1 7DN, UK; 2 Department of Physiology, Faculty of Health Sciences, University of Pretoria, Arcadia, 0007, South Africa

**Keywords:** Dormancy, persisters, sepsis, microbiome, inflammation, culturability, iron dysregulation

## Abstract

For bacteria, replication mainly involves growth by binary fission. However, in a very great many natural environments there are examples of phenotypically dormant, non-growing cells that do not replicate immediately and that are phenotypically ‘nonculturable’ on media that normally admit their growth. They thereby evade detection by conventional culture-based methods. Such dormant cells may also be observed in laboratory cultures and in clinical microbiology. They are usually more tolerant to stresses such as antibiotics, and in clinical microbiology they are typically referred to as ‘persisters’. Bacterial cultures necessarily share a great deal of relatedness, and inclusive fitness theory implies that there are conceptual evolutionary advantages in trading a variation in growth rate against its mean, equivalent to hedging one’s bets. There is much evidence that bacteria exploit this strategy widely. We here bring together data that show the commonality of these phenomena across environmental, laboratory and clinical microbiology. Considerable evidence, using methods similar to those common in environmental microbiology, now suggests that many supposedly non-communicable, chronic and inflammatory diseases are exacerbated (if not indeed largely caused) by the presence of dormant or persistent bacteria (the ability of whose components to cause inflammation is well known). This dormancy (and resuscitation therefrom) often reflects the extent of the availability of free iron. Together, these phenomena can provide a ready explanation for the continuing inflammation common to such chronic diseases and its correlation with iron dysregulation. This implies that measures designed to assess and to inhibit or remove such organisms (or their access to iron) might be of much therapeutic benefit.

## Introduction

“It is now well established that some micro-organisms can, under certain conditions, be deprived of all visible signs of life and yet these organisms are not dead, for, when their original conditions are restored, they can return to normal life and activity” ^1^.

“Bacterial populations in both batch and continuous culture are much more heterogeneous than is normally assumed, and such cultures may consist of several types of subpopulations simultaneously differing in viability, activity and integrity of the cells” ^2^.

**Figure 1.**
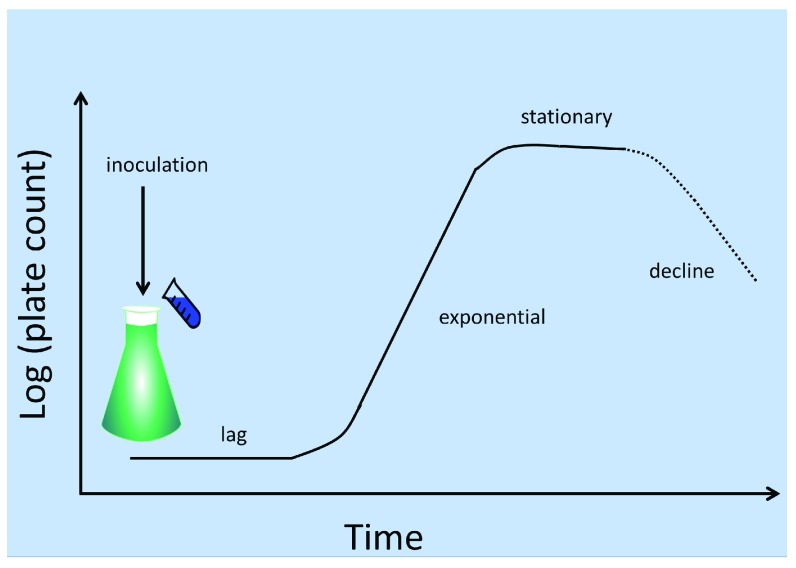
A typical laboratory bacterial culture. After the end of stationary phase the viable count decreases over time, but very rarely to precisely zero. Some authors recognise an extended “period of prolonged decrease” ^852^ during which some of the survivors undergo significant dynamics, and in which mutants are selected. Our interest here is largely in cells that have not mutated.

Consider a typical axenic flask or broth culture of bacteria ( Figure 1), arguably the staple of modern laboratory microbiology. We seed a suitable growth medium with an appropriate inoculum of cells known to be capable of replicating in that growth medium. After a lag phase the number of culturable cells (the ‘viable count’ ^3, 4^, as judged by plate counts of the number of colony-forming units observable on the same medium solidified by agar or a similar material) is observed to increase, typically exponentially, for a number of generations (the growth phase or exponential phase). Apart from the changes in nutrient concentration, and for non-synchronised cultures, it is generally taken that cells pass smoothly through their cell cycles *en route* to doubling their numbers by binary fission. The population distribution of organisms in different parts of their cell cycle during the exponential phase is thereby unchanged and thus in a steady state (from which the cell cycle parameters can even be inferred ^5^). In time this increase in cell numbers ceases, usually because of the exhaustion of a nutrient in a closed system, or sometimes in part or whole because of the build-up of toxins. Again, after a further period, the viable or colony count decreases (often to quite low levels if such starvation is carried out for extended periods). Inoculation of a new broth culture with a similar number of viable cells from this culture usually provides a simple repeat of the previous culture ^6^, and in the absence of mutation may reasonably be anticipated, for organisms proliferating asexually, to be played out indefinitely.

The development of continuous ^7^, nutrient-limited (‘chemostat’ ^8^) or feedback-controlled (‘turbidostat’ ^9– 11^) cultures was and is entirely consistent with this view of steady-state microbial doubling via homogeneous cell cycles that are common, within statistical fluctuations, to each cell. The same is true for cultures undergoing serial transfer (where there is slightly more of a focus on selection for genotypic variants that grow faster – see e.g. 12– 14).

**Figure 2.**
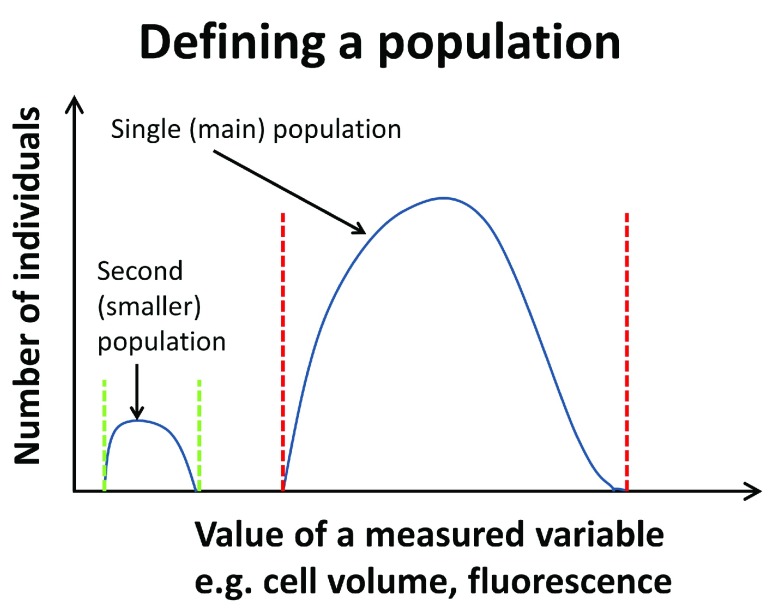
To clarify the general concept of a population as used here, a population of individuals involves those who share certain properties (between stated values). One main population is shown. A second, smaller population is also shown; these might represent dormant cells.

**Figure 3.**
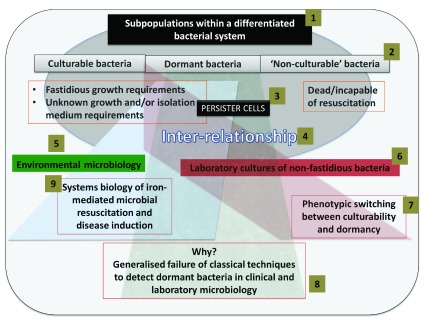
Infographic summary of the review. (1) A bacterial system contains distinct subpopulations, that we classify as culturable, dormant and non-culturable (2). Specific attention is given to persister cells (3), and the inter-relationship (4) between the subpopulations. Subpopulations within environmental biology are discussed (5), followed by subpopulations within laboratory cultures (6). Particular emphasis is placed on phenotypic switching between the culturable and dormant subpopulation of laboratory cultures (7). Generalized detection techniques typically fail to detect dormant cells, and we review the various reasons for this failure and discuss alternatives (8). Resuscitation of and endotoxin production by such dormant cells underpins many diseases not normally seen as having a microbial component.

**Figure 4.**
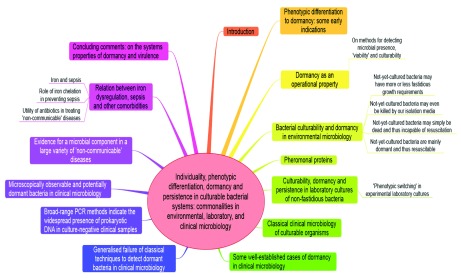
Summary of the review in the form of a ‘mind map’ ^853^ of the article.

There should be nothing controversial in the above passage, but in fact it hides a variety of assumptions that themselves conceal a considerable feast of very interesting physiology. The chief one here is that – given that all cells in the culture are genetically homogeneous and see the same ‘environment’, and *modulo* where they are in their cell cycles – all such cells are indeed supposed to represent a single population (as per Figure 2). If they do not, and as we shall see they never do ^15– 18^, we are dealing with differentiated systems. It turns out that a particular subset of typical cell cultures – a phenotypically dormant or non-growing sub-population, occurring even in non-sporulating bacteria ^2^ – is widespread to the point of ubiquity. This leads to an exceptionally important biology with significant consequences both for our understanding of microorganisms and our ability to harness and domesticate them. Although the relevant literatures rarely cite each other or overlap, it is clear that similar phenomena are common to bacterial behaviour in the natural environment, the laboratory, and in a variety of samples of clinical interest. This theory or hypothesis that we develop here comes about from the synthesis ^19^ of a large amount of data, and is summarised in Figure 3 and Figure 4.

## Phenotypic differentiation to dormancy or persistence – some early indications

**Figure 5.**
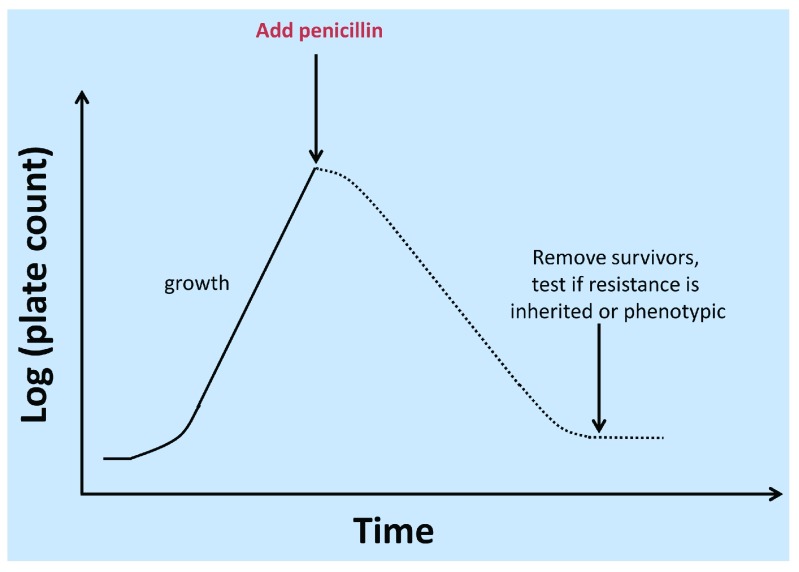
Assessment of phenotypic differentiation of a dormant subpopulation via antibiotic challenge. This kind of protocol can be used to determine if the resistant subpopulation has accumulated genetic mutations that encoded resistance or whether, as focused on here, the resistance is purely phenotypic. A detailed analysis of the shape of the time-survivor curves may also be informative ^854^.

While dormancy and resuscitation of rotifers had been observed by Leeuwenhoek himself in 1702 ^1^, some of the earliest modern indications for a physiologically significant ‘phenotypic heterogeneity’ ^20^ or differentiation of microbial cultures came in the 1940s. In a conceptually simple experiment (illustrated in Figure 5), Bigger ^21^ exposed staphylococcal cultures to concentrations of penicillin that would normally be sufficient to kill them completely (and they did kill all but 1 in a million). However, these (10 ^-6^) survivors, that Bigger ^21^ and McDermott ^22^ (and many modern commentators have) referred to as ‘persisters’, were not genetic mutations selected for resistance to penicillin, since when they were inoculated into fresh broth they were just as susceptible as were those in the first culture. Bigger recognised (correctly) that the only explanation that made any kind of sense was that despite being exposed to nominally the same conditions, these cells were operationally dormant in the sense of not replicating in a medium that, apart from the penicillin, would normally admit their growth (even if they were metabolically active ^23, 24^) and thus phenotypically resistant to the penicillin (that anyway kills only dividing cells ^25– 27^). Similarly, Luria and Latarjet ^28^ noted that approximately 1% of the cells in a culture of *Escherichia coli* displayed a phenotypic resistance to normally sterilising doses of ultraviolet irradiation. Many similar experiments since (e.g. 29– 32), discussed in more detail below, have recapitulated this basic phenomenon. (We note here that high-frequency antigenic ‘phase’ variation can occur due e.g. to changes in microsatellite DNA ^33^; detailed discussions of such genotypic changes ^34^, including those that can affect the extent of dormancy in persistent bacteria ^35^, are outwith the scope of the present, purely phenotypic analyses.)

## Dormancy as an operational property, and semantic issues

For the avoidance of doubt, and in accordance with Keilin’s description with which we opened, we shall define dormancy as:

“a reversible state of {often} low metabolic activity, in which cells can persist for extended periods without division; we shall see that this often corresponds to a state in which cells are not ‘alive’ in the sense of being able to form a colony when plated on a suitable solid medium, but one in which they are not ‘dead’ in that when conditions are more favourable they can revert to a state of ‘aliveness’ as so defined” ^2^.

We thus stress ^36^ the recognition that dormancy is not solely an innate property of a bacterial cell; it is a property assessed by one or more experiments, so whether a cell appears to be dormant depends on both the cell and the experiment used to assess that dormancy. (This principle shares a similar philosophical foundation to the independence from any specific experiment, or otherwise, of the perceived state of objects within the quantum theory ^36– 38^). As do Postgate ^3, 4, 39^ and Barer ^40– 44^, we take the hallmark of a viable or living bacterial cell to be its ability to replicate or its ‘culturability’. This means that we cannot tell via culturability that a cell is alive, only (after a cell division) that it was alive ^36, 45^. Dormant cells – even if ‘not immediately culturable’ – must by definition be resuscitable to form culturable cells. We also recognise (as does Michael Barer ^889^) that it may be hard to discriminate the resuscitation of dormant cells from the recovery of injured cells. Although the term ‘nonculturable’ is quite commonly used to describe not-immediately-culturable cells it is best avoided, as we cannot try every possible combination ^46^ of incubation conditions that might serve to resuscitate a cell in a sample. ‘Non-cultured’, ‘as-yet-uncultured’ or ‘operationally nonculturable’ are better terms. Culturable, (operationally) non-culturable and (operationally) dormant bacteria in the differentiated bacterial (cellular) system can therefore be seen as distinct subpopulations of the system, and culturable and dormant bacteria as reversible states of the same population. A culture containing several subpopulations, whether distinct (as in Figure 2, or part of a single population characterised by a particular value from a range of an extensive variable) may be said to be differentiated (and of course may de-differentiate) in terms of physiological macrostates, that may or may not be able to interconvert. However, we recognise (thanks to Michael Barer ^889^) that such interconversion does not imply a mechanistic reversibility. The same kinds of issues attach to cells described as having any other physiological property with regard to the ability to replicate. We note (with thanks again to Michael Barer ^889^) that it is easy to conflate dormancy and ‘persistence’, since they do share some similarities (e.g. such cells are not immediately replicable); however, there is not much in the way of evidence as to how different say their expression profiles are, since it would require, for instance, single cell omics measurements, that are only just becoming available (e.g. 47, 48), more typically ^49^ for the much larger eukaryotic cells. Certainly there can be extensive changes in gross biochemical composition as cultures are starved ^50^. One strategy would be to separate sub-populations ^51, 52^, acquire ‘averaged’ values of say their transcriptome, proteome or metabolome, and see how much they differed. In a similar vein, whether states such as dormancy are adaptive is a matter for experiment.

**Figure 6.**
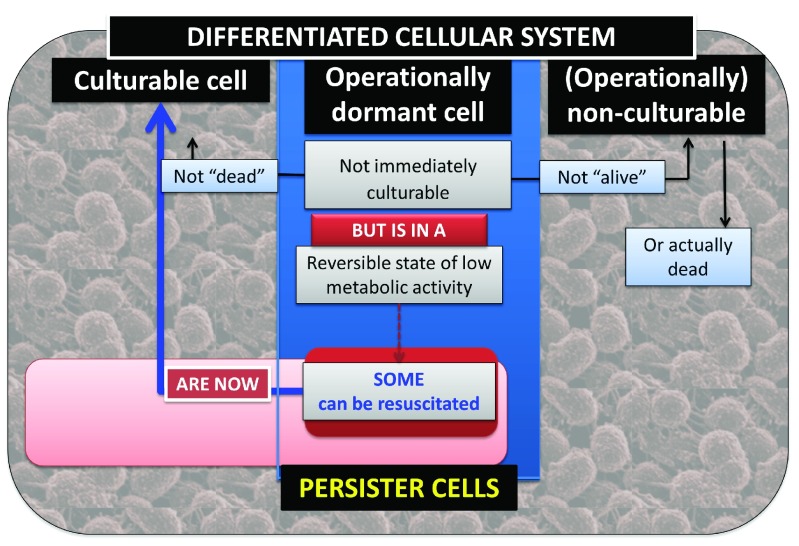
The relationships between culturable, dormant and non-culturable bacteria within a differentiated cellular system.

The general relationships between various subpopulations of the bacteria within a differentiated cellular system are shown in Figure 6.

### On methods for detecting microbial presence, ‘viability’ and culturability

Given our operational definition of dormancy as including reversible culturability, we note that different kinds of assays for the presence or activity of bacteria necessarily reflect cells in different kinds of physiological states (and can thereby be used to discriminate them). Thus direct counts with stains such as acridine orange (a list of these and other methods is given in Table 1 of 36) do not determine culturability, only presence or activity. Similarly, macromolecular sequencing methods such as those based on rDNA and its amplification (e.g. 53– 58), or that of other housekeeping genes (e.g. 59– 61), almost certainly reflect mainly dormant cells plus any actively dividing ones (in that ‘naked’ DNA is usually degraded fairly rapidly in serum or the environment). The difference between culturable counts and total sequence-based counts probably provides one of the best methods for detecting and enumerating potentially dormant cells when they cannot yet be brought back into culture, although (as recognised by referee 1) such differences may also reflect dead, injured or moribund cells. It is particularly noteworthy (and see also 62 and below) that the amount of prokaryotic DNA in whole blood exceeds by 10–100-fold that detectable in serum ^63^, implying adsorption onto or sequestration within blood cells.

We shall return to clinical and laboratory microbiology later, but it is to environmental microbiology that we now turn to discuss the culturability of typical microbes. While the same general truths undoubtedly pertain in viruses (e.g. 64, 65), and in yeasts, fungi, archaea, mycoplasmas and other unicellular organisms, our focus will be on prokaryotes.

## Bacterial culturability and dormancy in environmental microbiology

It has long been known that the number of bacteria observable microscopically exceeds, typically 100-fold, those that can readily be grown axenically in standard isolation media (i.e. to proliferate in liquid culture or to form colonies on solid media). The latter has been referred to as ‘the great plate count anomaly’ ^66^, and has been amply confirmed by more modern, culture-independent sequencing methods. A selection of papers and reviews serve to document both the numerical anomaly and the much greater biodiversity detectable by sequencing (e.g. 67– 86). It is thus useful to discriminate (1) bacteria that have been cultured, that are typically available in culture collections, and whose growth requirements are known, from (2) bacteria that may be recognised as novel via macromolecular sequencing (typically of ribosomal DNA ^80, 87– 90^) but that have not yet been cultured and whose growth requirements may not yet even be known. Much (sequencing) evidence indicates that the bulk of the ‘missing microbes’ or ‘dark matter’ ^91– 93^ in natural ecosystems falls into this second category ^94^, and that ‘single cell’ methods may be required to culture them ^95^.

There are at least four general reasons of principle why these organisms have not yet been cultured. We consider each in turn (although more than one may contribute in individual cases).

### Not-yet-cultured bacteria may have more-or-less fastidious growth requirements

It is an elementary observation in microbiology, and the basis for selective isolation media, that not all bacteria grow on all media and in all conditions. Leaving aside truly syntrophic bacteria (that for thermodynamic or unknown nutritional reasons require another organism for growth (e.g. 96– 102)), some organisms may have quite fastidious growth requirements. A number of bacteria determined as causative of disease, whose role had originally been inferred only through microscopic observation, were later cultured and could be shown to fulfil Koch’s postulates. These include *Helicobacter pylori* ^103, 104^ (with an unusually high requirement for urea to fuel its alkalinogenic urease activity ^105^) and *Legionella pneumophila* ^106– 109^ (with an unusually high requirement for cysteine). Note that even the supposedly rich LB medium ^110^ (Lysogeny Broth, often erroneously called Luria-Bertani medium, see http://schaechter.asmblog.org/schaechter/2009/11/the-limitations-of-lb-medium.html) is not in fact a particularly rich medium ^111– 113^. An especially nice example ^114, 115^ is provided by *Tropheryma whipplei*, the causative organism of Whipple’s disease ^116, 117^. It resisted attempts (over many decades) to bring it into axenic culture until systematic genome sequencing ^118, 119^ showed its requirements for a variety of common amino acids that it was unable to synthesise itself, the provision of which permitted its growth. The MetaGrowth database ^120^ is now available for similar purposes. Another good example is *Coxiella burnetii*, the causative agent of Q fever, for which a genome-derived growth medium (‘acidified citrate cysteine medium’) permitting axenic culture has now been developed ^121, 122^. Other examples are given by Stewart ^123^ and by Singh and colleagues ^114^, and include marine bacteria of the highly common SAR11 clade ^83, 124, 125^. Of course these kinds of phenomena are not absolute; much evidence indicates that host stress hormones may act as growth or virulence factors for a variety of Gram-negative organisms, representing a kind of ‘microbial endocrinology’ (e.g. 126– 128).

### Not-yet-cultured bacteria may even be killed by our isolation media

Organisms in nature are often living in low-nutrient conditions ^129– 133^. It is thus reasonable (and unsurprising) that the isolation of microbes from starved, oligotrophic environments benefits from the use of low-nutrient conditions ^75, 123, 134– 136^; some manifest this ‘starvation’ through their size, as ‘ultramicrobacteria’ (see e.g. 137– 143). In a similar vein, taking cells from low-nutrient natural environments directly onto, say, a highly aerobic agar plate may produce stresses that effectively kill them, so that afterwards they would not even grow on the kinds of media (as in the previous section) that would support their growth. Thus, Tanaka and colleagues ^144^ showed interactions between phosphate and agar when autoclaved together that led to the production of compounds inimical to bacterial growth. Gellan may be a better solidifying agent here ^96^. However, we recognise that it may be hard to discriminate cells that we kill in the act of trying to isolate and grow them from ‘already dead’ bacteria.

### Not-yet-cultured bacteria may simply be dead and thus incapable of resuscitation

While this possibility certainly exists, and is included for completeness, it is actually the least likely for a number of conceptual and empirical reasons. The first is that if an organism is present in a particular environment it must have been able to grow and divide in it at some point in the more or less recent past, even if the result of such growth was its utilisation of a finite amount of necessary nutrients or growth factors whose exhaustion caused replication to cease. (Interestingly, in soil it seems that sequestration, rather than complete exhaustion, of nutrients is the more significant phenomenon ^145– 147^.) Secondly, it is highly unlikely that evolution could select for unicellular organisms that cannot replicate. Thirdly, environmental organisms can be shown to metabolise even when they cannot be shown to divide (e.g. in the ‘Direct Viable Count’ method ^148^ and in any number of other tests that detect metabolic activity ^36, 149^). And finally, as we shall see in the next section, careful methods of resuscitation/cultivation do indeed allow a very significant fraction of organisms that can be isolated from a variety of environments (e.g. the gut ^150– 153^) to be resuscitated and to grow very effectively.

### Not-yet-cultured bacteria are mainly dormant and thus resuscitable

As indicated in the introduction, it is now well established that even laboratory cultures, that from a macroscopic point of view are growing exponentially, contain subpopulations of non-growing cells. These cells are dormant by definition, because they may later be resuscitated and grow. It is easy to ascribe an evolutionary advantage of this culture differentiation from the perspective of the benefits of having a sub-population that by not growing is more resistant to environmental stresses (e.g. 154– 156). Indeed, this general kind of phenotypic differentiation strategy, in which the variance in reproductive rate is traded off at the expense of the mean, has been referred to as bet hedging ^78, 156– 167^ and is actually adaptive ^168, 169^. An important point here ^168^ is that in many natural environments, asexually reproducing organisms such as bacteria are likely to be (spatially) close to their ancestors and descendants, such that inclusive fitness theory ^170, 171^ implies that it is entirely reasonable for them to behave altruistically, e.g. by ‘bet hedging’. This is also discussed further below.

It is also reasonable that in isolated (closed) natural environments, nutrients and thus sources of energy must be exhausted at some point, and thus for simple energetic reasons multiplication becomes impossible and a dormant state likely (if later resuscitation proves it to be so). Similarly, it is likely that in the absence of energy, nutrients and/or signalling molecules, and based on more ecological or community considerations (e.g. 172– 175), it is necessary to add any or each of them to ‘prime’ bacteria to resuscitate. This has indeed been shown ^70, 174, 176– 179^, including for sources of energy ^180, 181^, iron-acquiring compounds ^182^ (siderophores ^183– 185^), cell wall muropeptides ^186^, and various signalling molecules ^187, 188^ (especially pheromones ^168, 169, 189, 190^) that exist in natural environments ^70, 174, 191^. We note too that ‘kick starting’ dormant cells may require the synthesis of transporters (a neglected clade ^192^) necessary for the uptake of all kinds of molecules ^193– 197^. Overall, the idea that most bacteria that may be observed in the natural environment are ‘unculturable’ is incorrect.

Finally here, and though this is obvious it is well worth rehearsing, the simple fact that we can store non-growing microbes under desiccated or frozen conditions or as agar ‘stabs’ in culture collections for extended periods means that most microbes are certainly well adapted to entering and leaving dormancy.

## Pheromonal proteins

A related and unexpected discovery came from analyses of starved laboratory cultures of the actinobacterium *Micrococcus luteus*, in which almost all cells lost culturability ^2, 198– 200^. However, they were not dead but dormant, as they could be resuscitated by using a combination of weak nutrient media and a signalling molecule found in spent culture supernatants ^201– 206^. The original studies used flow cytometry to discriminate the physiological state of individual cells ^51, 207– 210^ (see also 211, 212). By using another ‘single cell’ assay based on dilution to extinction (that avoids artefacts connected with the regrowth of ‘initially viable’ bacteria ^36^), we were able to purify the signalling molecule. It turned out to be a protein, named Rpf (for ‘resuscitation-promoting factor’) ^213^. In *M. luteus* there is only one homologue ^214^, and the gene (product) is essential for both resuscitation and multiplication ^213, 215^. Rpf contains a highly conserved 70 amino acid ‘Rpf domain’ and is widely (and probably ubiquitously) distributed throughout the actinobacteria ^216– 219^, but with examples elsewhere ^220, 221^. Most organisms that have a homologue have more than one. Thus *M. tuberculosis* has five homologues ^222– 224^. Rpfs can have peptidoglycanase and muralytic activity ^225– 230^ and known crystal structures are consistent with this ^231– 236^. These activities can certainly account for at least some ^237^ of the resuscitation-promoting properties. As an extracellular protein that may be required for growth, and with a high level of immunogenicity ^238^, it is obviously an excellent candidate target for inclusion in appropriate vaccines against pathogenic actinobacteria ^213, 225, 239– 246^. It is also more directly of potential utility in stimulating bacterial communication and resuscitation in a variety of cultures in both samples taken from Nature ^247– 257^ and in the laboratory ^258– 271^.

## Culturability, dormancy and persistence in laboratory cultures of non-fastidious bacteria

Having established the frequency of occurrence of microbial dormancy in the natural environment, it is of interest to understand better the mechanisms by which microbes might effect this dormancy and potential resuscitation. Unsurprisingly, microbiologists have turned to *E. coli*, and considerable progress has been made ^24, 272– 279^.

The starting position is as in Figure 1 and Figure 6, to the effect that at any given moment in a typical culture a small fraction of the population is non-growing, and thus potentially dormant. Since clearly the same fraction cannot (or is wise not to) remain in dormancy indefinitely in the presence of suitable nutrients that permit the growth of its siblings, we must invoke at least one mechanism that can cause the bacteria to ‘oscillate’ between growing and dormant states. Many simple gene expression network topologies admit this behaviour ^159, 280– 284^, including a simple feedback loop with delay ^285, 286^, and we note that even whole cultures can exhibit oscillations and deterministic chaos ^287^. While flow cytometric observations (e.g. 51, 288) show that even ‘homogeneous’ laboratory cultures show highly heterogeneous distributions in cellular volume (not just between X and 2X) and expression profiles (and see 289), our particular focus will be on ‘binary’ or ‘bistable’ systems in which individual cells either are or are not operationally culturable.

Experimentally, it is also common to assess the phenotypic ability of subpopulations of cells to tolerate normally inhibitory concentrations of bactericidal drugs ^290, 291^, this being a marker for that fraction of cells that is ‘persistent’ (and maybe dormant) at the stage in question. Note that the persistence phenotype is not induced by the drugs ^275^. Changes or transitions in the state of a particular cell in a population between the various phenotypic states is a phenomenon that may be (and is commonly) referred to as ‘phenotypic switching’.

### ‘Phenotypic switching’ in experimental laboratory cultures

A particularly well-developed example of this ‘bet hedging’ or phenotypic switching between physiologically dormant and growing states may be observed in laboratory cultures of organisms such as *E. coli* demonstrating ‘persistence’ ^161, 164, 166, 292– 298^. In general, any scheme in which both a first gene product inhibits cellular proliferation and in which this first gene product may be titrated out potently ^299^ by a second gene product that thereby undoes the inhibition of proliferation, can have the effect of phenotypically switching cells between dormancy and growth. This seems to be precisely what is going on, and such pairs of gene products have been referred to (somewhat misleadingly ^300^) as toxin-antitoxin (TA) pairs ^300– 307^. One such involves the well-known pp(p)Gpp metabolic system that can serve to inhibit DNA gyrase ^24, 308– 311^, and points to the fact that in these circumstances, persisters may be quite metabolically active ^23, 24, 309, 312^, even if transiently incapable of reproduction. Another phenotype switching mechanism, underlying colony phenotype switching, comes from metabolic bifurcations driven by the levels of a particular metabolite ^313^.

Any mechanisms that permit cells to communicate with each other can amplify switching effects by cell synchronisation, and by definition such ‘social’ signals act as pheromones, whose apparent ‘altruism’ can be explained on the basis of kin selection theory ^168^. There is considerable interest, largely outwith our scope here, in these evolutionary aspects (e.g. 314– 321). Such systems are commonly, but far too broadly relative to the term’s origin ^322^, referred to as ‘quorum-sensing’. However, they do offer opportunities for limiting bacterial virulence (e.g. 323– 330).

## Classical clinical microbiology of culturable organisms

Until relatively recently, almost all of clinical microbiology ^331, 332^ was based on rather classical methods of plate counting ^333^, coupled to assessment of antibiotic sensitivity. Various means of automated blood culture that assess metabolism exist (although they require typically 48–72h to show a ‘positive’) ^334^. Positive tests, often implicitly involving culture (and not just metabolism) within the assay, would be followed by other tests seeking to identify the organisms detected, nowadays typically by nucleic acid sequence-based methods ^58, 335– 338^. However, these and other tests for the presence of antigens or even antibodies ^339^ cannot speak to the question of culturability (and of course antigens such as lipopolysaccharide (LPS) are shed by dying cells). This said, it makes little sense to try to culture microbes from samples that molecular sequencing methods indicate lack them, so the molecular methods always provide a useful starting point for seeking to resuscitate any resuscitable (hence operationally dormant) microbes that might be present.

The existence of bacterial DNA in even ‘healthy’ blood has long been known ^340^, and since naked DNA would be degraded and living cells would soon kill the host, the (seemingly) obvious conclusion that the prokaryotic DNA must reflect occult, and potentially dormant, cells seems neither to have been drawn nor acted upon.

## Some well-established cases of dormancy in clinical microbiology

The idea that (typically intracellular) dormancy is a major component in some infectious diseases (including in the absence of antibiotics that may serve to light up ‘persisters’) is of course well-established, and the main purpose of this brief section is simply to remind readers of this. Such a reminder serves as a prelude to a longer discussion of the very many clinical circumstances where we consider that the role of dormant microbes is not widely appreciated, and where they are not really considered to involve a communicable or microbial component at all. Thus Table 1 shows a few organisms (and references) for which we consider that most readers would regard the idea of and evidence for dormancy as more or less uncontroversial. We do not include disease-causing infectious agents where they are better known for their ability to persist in the natural environment. Organisms such as *Legionella pneumophila* that represent significant public health issues, fall into this category, and *Legionella* and other persisters (in environments such as water system biofilms) are indeed well known (e.g. 341– 345), although they too have special adaptations to an intracellular lifestyle (e.g. 346).

**Table 1.** Some bacterial infections for which an intracellular, reversibly non-replicating, persistent or dormant state is well established as part of the cells’ lifestyle. Examples are given for both low- and high-GC Gram positives, as well as a number of Gram-negative organisms.

Organism	Comments	Selected references
*Bartonella* spp.	Persists inside erythrocytes	347– 350
*Brucella* spp.	Environmental and intracellular persistence and immune evasion	351– 354
*Listeria monocytogenes*	Well-established low-GC Gram-positive intracellular saprophyte and non- sporulating persister	355, 356
*Mycobacterium tuberculosis*	Often seen as the ‘classical’ dormant bacterium, a high-GC Gram-positive; probably one third of humans carry it in a latent or potentially dormant state; other forms may be metabolically active	357– 366
*Salmonella typhimurium*	Gram-negative; non-replicating forms common in macrophages and elsewhere	367– 370
*Staphylococcus aureus*	Low-GC Gram-positive; can escape antibiotics by hiding inside various phagocytes	371– 374

## Generalised failure of classical techniques to detect dormant bacteria in clinical microbiology

As noted above for environmental microbiology, dormant bacteria can represent as much as 99% of the organisms that may be observed microscopically or by macromolecular sequencing, but classically (and by definition) they are not enumerated by culture-based methods that determine ‘immediate culturability’ ^36^. Such culture-based methods are also widely used in clinical microbiology. However, if we were to plate out 100 μL of a culture containing 200 bacteria.mL ^-1^, of which 99% were dormant at any instant, we would expect (based on a Poisson distribution) to see fewer than 1 propagule or colony-forming unit per sample. We have noted above that it can be determined by sequencing that many of the non-cultured environmental organisms largely differ from those in standard culture collections. Certainly the examples given above in clinical microbiology, such as *Tropheryma whipplei*, were both observed microscopically and were sequenced prior to being brought into axenic culture.

The PCR method is exquisitely sensitive (down to one cell or propagule per sample), and we note that contamination artefacts from the PCR reagents represent a real issue that must always be checked (e.g. 375– 379), albeit this is no less true of blood cultures ^380^. We have rehearsed elsewhere ^62^ five classes of argument that collectively make it implausible that these are all contamination artefacts; probably the most persuasive is simply the sheer number of prokaryotic DNA molecules that can be measured in blood and serum (e.g. 381– 383). While some of the most recent nucleic acid sequencing methods (e.g. 384– 389) do operate on single molecules, and genome-wide sequencing may soon be routine (e.g. 390, 391), the analysis of prokaryotes usually used a broad-range PCR step to amplify small-subunit rDNA to assess their presence, whether in environmental ^74, 80, 88, 392^ or clinical ^388, 393– 405^ samples. Using this, and while these methods alone cannot tell whether they were operationally dormant or dead, a very considerable number of studies have been performed in which ‘culture-negative’ clinical samples showed the presence of prokaryotes (at least as judged by sequence-based methods). This has some profound consequences.

We note that in a steady state such cells must be supplied at a rate equal to that of their clearance, and that the fact that clearance is lower than probably expected implies a significant ability of such cells to evade the innate and adaptive immune systems. We also take it that at least for common organisms (not very slow growers such as certain mycobacteria) the former rates must be much lower than those typically attainable in laboratory cultures, else we would have classical sepsis, and we do not. Most likely the observable facts are best accounted for by a combination of a periodic resupply of resuscitating cells, coupled to physiological changes in non-growing cells (especially including of cell wall antigens) that help them evade natural clearance mechanisms.

## Broad-range PCR methods indicate the widespread presence of prokaryotic DNA in culture-negative clinical samples

While PCR-based methods have long been used to assess the species involved in culture-positive samples ^406^, e.g. from blood, our interest here is in samples that are culture-negative ^407^ that may yet (and indeed likely do) contain dormant cells. Among the first such indications of this was the study by Relman’s group ^340^, who showed that the blood of even healthy controls contained significant amounts of prokaryotic DNA. Table 2 lists some studies in which broad-range PCR has been used to amplify and detect prokaryotic rDNA in culture-negative samples.

**Table 2.** Some examples of blood culture-negative but PCR-positive systems, implying the presence of dormant bacteria. Note that we have sought to exclude examples where anaerobic bacteria could be detected by PCR but not cultured simply because cultures were not anaerobic, and also cases (e.g. 408, 409) where high antibiotic concentrations might have prevented culture.

Aims	Culture-negative but PCR-positive	Reference
Assessment of endocarditis	6 out of 29	410
Development of broad-range PCR	71 out of 382	406
Development of broad-range PCR; limit of detection 5000 cfu.mL ^-1^	10 out of 103	411
Improved broad-range PCR method	20 out of 24	53
Review	Many examples	412
Interstitial cystitis	14 out of 14	413
Endocarditis	270 (36.5%) of 740	414 (and see 415)
Endophthalmitis	116 out of 116 (selected)	416
General study	18 out of 394 (271 also culture-positive, PCR-positive)	417
Bacteraemia in intensive care	48 out of 197 45 out of 94	418 419
Sepsis/SIRS	29 out of 59 38 out of 72 culture-positive 14.6% vs 10.3% (no antibiotics) 123 vs 95	420 421 422 423
Osteoarticular samples	141 out of 1667	424
Review	Many examples	425
Various, including antibiotic-treated	34 out of 240	426
Meningitis	26 out of 274 19 out of 21	427 428
Orthopaedic samples	9% out of 125	398
Thoracic empyaema	14 out of 22	429
Trauma	28 out of 35	430

In environmental microbiology, as mentioned above, there were many early indications (as observed microscopically or flow cytometrically) for the presence of bacteria that did not (or not easily) prove resuscitable or culturable. In a similar vein, many studies have shown microscopically observable organisms in culture-negative but disease-positive samples. This is true both for diseases considered to be due to microbial pathogens and, in fact, for many others normally considered non-communicable ^62^.

## Microscopically observable and potentially dormant bacteria in clinical disease

Microscopic observations in tissues have been a major part of the discovery process by which certain bacteria were indeed identified as the cause of various diseases. Billings ^431^, Price ^432^, Domingue ^413, 433– 435^, Mattman ^436^, Ewald ^437^ and Onwuamaegbu and colleagues ^438^ review the extensive and largely forgotten early literature. Domingue and Schlegel ^439^ also mentioned that they could recover culturable bacteria, probably mainly from L forms (see 62, 436, 440), from lysates of normal and diseased blood. It was to be assumed that these cells were not replicating at significant rates in the blood itself. However, we can find no evidence that this was ever followed up. Our own work ^441, 442^, summarised in 62, showed that both bacillary and coccoid cells could be found attached to and within the erythrocytes of patients with Parkinson’s disease and Alzheimer’s disease, at rather greater concentrations than in samples taken from nominally healthy controls.

**Figure 7.**
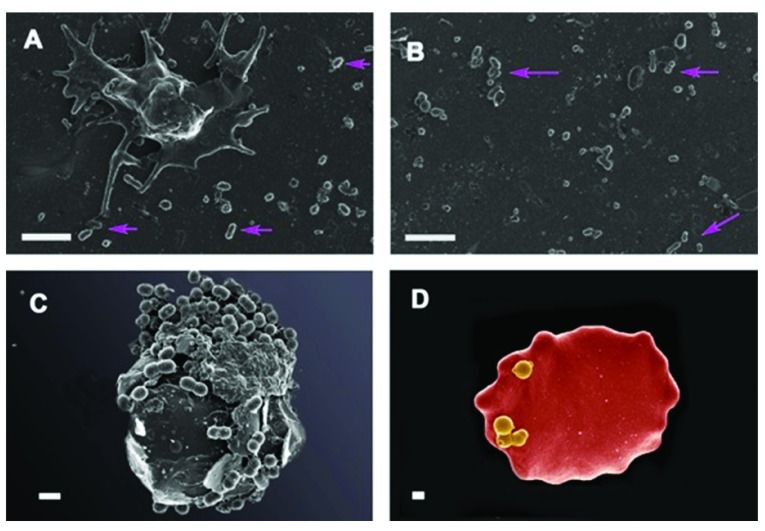
**A** and **B**) Platelet rich plasma (PRP) from a patient with systemic lupus erythematosus (SLE). **A**) Platelet with bacteria visible in the surrounding smear (pink arrows); **B**) areas in smear with bacteria (pink arrows); **C**) Erythrocyte with associated bacteria from patient with confirmed hereditary hemochromatosis **D**) Erythrocytes with bacteria from patients with diagnosed type II diabetes. **A**– **C** Scale bar: 1 μm and **D** 400 nm.

**Figure 8.**
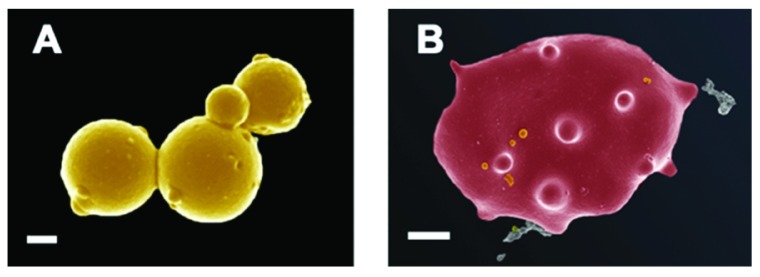
Bacteria in whole blood from a patient with thromboembolic ischemic stroke **A**) Microbiota in whole blood; scale bar: 200 nm. **B**) Erythrocyte with bacteria; scale bar: 1 μm.

In a similar way, our preliminary data show that bacteria are visible in plasma, as well as in whole blood smears in various inflammatory conditions. Here we show bacteria in platelet-rich plasma (PRP) taken from a patient with systemic lupus erythematosus and smeared onto a glass cover slip ( Figure 7A and Figure 7B). We also show the same from patients with hereditary hemochromatosis ( Figure 7C) and type 2 diabetes ( Figure 7D). We also noted microbiota associated with erythrocytes in thromboembolic ischemic stroke ( Figure 8A and Figure 8B). (Our microscopy methods are as published previously (e.g. 442– 451), but fuller publications will appear elsewhere). The ultramicroscopic evidence that these are indeed small bacteria and not say, cellular debris or microparticles (see 452) is presently mainly morphological, though we note the considerable evidence for the presence of bacterial DNA in blood (see previous sections and e.g. 63, 340, 453).

It is worth rehearsing the very great significance of this. With erythrocytes being present at some 5x10 ^9^.mL ^-1^ in human blood, even if only one erythrocyte in a thousand harboured just a single dormant bacterium (that would be hard to detect microscopically, but see 453– 457), the dormant bacterial load would still be 5,10 ^6^.mL ^-1^. This is both far from negligible, and serves to exclude the (always potentially worrisome) claim that ‘it is all contaminants’.

## A culturable blood microbiome

A recent and highly significant paper by Damgaard and colleagues ^458^ bears discussion. These workers note ^458^ that while bacterial growth can normally be elicited during sterility testing *in vitro* from fewer than 1 in a 1000 blood units ^459– 461^, transfusion-transmitted infections occur with a very much higher frequency (more like 10–12% ^462, 463^, or even more ^464^), and are responsible for a high fraction of transfusion-associated deaths ^465– 467^. Although it was acknowledged that venepuncture-associated contamination or an effect of transfusion in suppressing the immune system might contribute, it was also recognised ^458^ that one means by which to account for this would be that ‘normal blood’, and in particular its erythrocyte components, might also contain infectious agents that might be able to grow post-transfusion. Indeed, these authors found ^458^ that under anaerobic conditions a small number of colony-forming units (ca 4–5.mL ^-1^) could be recovered by direct plating from fully 62% of blood units, with ‘controls’ producing an average of just 1 cfu.mL ^-1^. More of the bacteria were associated with red blood cells than with plasma, and rDNA was used to identify them. These data are entirely consistent with the idea that dormant bacteria are present in the blood of even ‘normal’ individuals (note that periodontitis was not a criterion for donor exclusion here ^458^), that they are probably lurking in or on erythrocytes ^468, 469^, and that they can be resuscitated and grow under the correct conditions.

## Evidence for a microbial component in a very large variety of ‘non-communicable’ diseases

We have surveyed the literature for evidence in which a microbial component has indeed been observed to be an accompaniment of, and probably a major contributory factor to, a variety of (typically inflammatory) diseases that are normally considered ‘non-communicable’. Rarely has the physiological state of these microbes been considered, but since it would be obvious if they were growing, it is most likely that they are indeed dormant. Table 3 summarises these highly extensive associations. While some are just associations, and we could have extended this table considerably, some studies (e.g. 470) contain very detailed aetiological arguments that leave little room for doubt. Overall, the sheer size of the Table does strongly indicate the commonality of many of the microbially based mechanisms underpinning or accompanying various autoimmune and inflammatory diseases. In conditions such as atherosclerosis, transient ischemic attacks (TIAs), and stroke, it is very easy to conceive how resuscitating bacteria might serve to block the flow of blood, for instance. At all events, our main point here is that the evidence for a microbial contribution to many diseases supposedly lacking a microbial component is both multi-factorial and very considerable. Indeed, the purpose of a synthetic review such as this is to provide such pointers for more detailed studies in individual cases. Our specific interest is with the chief mechanisms by which these supposedly dormant bacteria might resuscitate and act as triggers of disease.

**Table 3.** Evidence for infectious agents in non-communicable diseases. We purposely largely confine ourselves to bacteria here, but include the occasional parasite, fungus, mycoplasma and virus. While obesity is usually seen as a cause of other diseases, rather than a disease itself, we note the influence of endotoxaemia on obesity ^471– 476^. We note too the extensive evidence for the role of LPS in inflammation ^477– 479^, and the experimental models (e.g. for Parkinson’s ^480^) where it can induce disease directly. We do not much discuss diseases such as Crohn’s disease where the extensive uncertainty over the extent of involvement of mycobacteria (e.g. 481– 483) needs no extra rehearsal (albeit it serves to illustrate the difficulties of identifying the role of hard-to-cultivate bacteria in chronic diseases). Further, while similar phenomena may be observed in a variety of cancers (e.g. 484– 489), for reasons of space we have determined that this must be the subject of a separate work.

Disease	Class of bacteria	Nature of the evidence of involvement	Selected References
AUTOIMMUNE DISEASES			
Ankylosing spondylitis	*Klebsiella pneumoniae*	LPS antibodies found in various patient populations	490– 493
Multiple sclerosis	*Clostridium perfringens*	Single case isolation: Immunoreactivity to ETX, fecal culture and PCR analysis, lysogenic bacteriophage footprint analysis (to exclude the possibility of laboratory contamination), sequencing of the patient-derived ETX gene	494
	*Chlamydia (Chlamydophila)* *pneumoniae*	17 patients with relapsing-remitting MS, 20 patients with progressive MS, and 27 patients with other neurological conditions. Bacterial present in the cerebrospinal fluid.	495– 501
	*Chlamydia (Chlamydophila)* *pneumoniae*	PCR, Serology Many patients studied: cerebrospinal fluid	496– 498, 500, 501
	Infectious causes of multiple sclerosis – discussion in The Lancet Neurology		499
Rheumatoid arthritis/ Osteoarthritis/reactive arthritis	*Porphyromonas gingivalis*	Periodontal bacterial DNA in serum and synovial fluid of many patient groups Anaerobic cultures (from subgingival samples), PCR, ELISA	502– 506
	*Porphyromonas gingivalis*	Antibody responses found in many patients	503, 505
	*Proteus mirabilis,* *Escherichia coli*	ELISA and indirect immunofluorescence techniques Anti-LPS antibodies and human serum Elevated levels of IgM and IgA specific to bacteria Studies involving many patients	470, 507– 515
	*Mycoplasma* ( *arthritidis* mitogen, *hominis* and *fermentans (MAM)*)	PCR, Western Blot Elevation of antibodies to MAM in RA sera: stuies involve many patients	520– 522
		Mycoplasma in 209 synovial fluid samples	520
	*Staphylococcus aureus*	Microbiology reports from patient records	523, 524
	*Salmonella* *Shigella* *Yersinia* *Campylobacter* *Clostridium difficile*	Review discussing the involvement of these bacteria in arthritis	525
	*Propionibacterium acnes*	In 23 of 55 patients, undergoing primary shoulder joint replacement, *P. acnes* was found in the joint fluid	526
	*Chlamydia trachomatis*	Synovial tissues of patients: review of literature	528
		*Chlamydia* from synovial fluid in single case	527
Systemic Lupus Erythromatosus	Cell wall-deficient form	Histologic observations of coccoid forms suggestive of cell wall deficient bacteria in cutaneous and systemic lupus erythematosus in 7 patients	529
	*Streptococcus pneumonia,* *Haemophilus influenza,* *Mycobacterium tuberculosis,* *Listeria monocytogenes,* *Klebsiella pneumonia,* *Staphylococcus aureus;* *Cryptococcus neoformans,* *Aspergillus fumigatus*	Blood & tissue culture, patient records Hypocomplementaemia and infection with encapsulated bacteria	530– 534
Vasculitis	Possibly mainly viral, but bacteria include *Staphylococcus aureus*, *Treponema pallidum,* Rickettsiaceae, *Borrelia burgdorferi*, *M. tuberculosis*	Various reviews that suggest bacterial involvement	535– 541
CARDIOVASCULAR DISEASES			
General	Comprehensive reviews		383, 542, 543
Atherosclerosis	*Aggregatibacter* *actinomycetemcomitans*	This was an animal (mice) study	544
	*Chlamydia (Chlamydophila)* *pneumoniae*	Antigens, PCR and treatment of patients with antibiotics with good results	545– 549
	*Helicobacter cinaedi*	This was an animal study. H. cinaedi infection significantly enhanced atherosclerosis in hyperlipidaemic mice	550
	*Helicobacter pylori* *Chlamydia pneumoniae*	Bacteria in atherosclerotic plaques of carotid arteries: PCR detection: study comprised 52 patients	547
	*Porphyromonas gingivalis*	PCR: periodontopathic bacteria were detected in atherosclerotic arterial wall specimens of large patient group	551– 556
		PCR, IgG Titers Against *P.gingivalis* Measurement	553
		Comprehensive reviews	554, 556
		PCR in a murine models	551, 555
	Periodontopathic bacteria *Prevotella intermedia* *Treponema denticola*	PCR: large patient based study	552
	*Streptococcus pneumoniae*	Inoculated animals	557
	*Toxoplasma gondii*	Animal (mouse) model	558
Endocarditis	Many cell-wall-deficient forms	Comprehensive review	559 See Table 2
		Benefit of antibiotic prophylaxis: review of literature	560
Hereditary haemochromatosis	*Chryseomonas, Veillonella,* *Streptococcus*	qPCR: 454 pyrosequencing of 16S rRNA genes to survey the bacterial diversity of atherosclerotic plaque, oral, and gut samples of 15 patients with atherosclerosis	561
	*Gemella haemolysans*	Blood culture (Gram stain, catalase activity and biochemical characteristics)	562
	*Listeria monocytogenes*	Letter to the editor regarding infection	563, 564
		Case study	564
	*Plesiomonas shigelloides*	Case study: Blood culture; API20E system	565
	*Vibrio vulnificus*	Case study: wound infection	566, 567
		Infected wild-type and hepcidin-deficient mice	567
	*Vibrio cholerae*	Case studies: Blood culture; PASCO and API20E	568
	*Yersinia enterocolitica*	Case studies: Microbial cultures, serotype O:3, serotype 9	569– 572
	*Yersinia pseudotuberculosis*	Case studies: Mobility test and API	573, 574
Hypertension	Periodontal infection with *A. actinomycetemcomitans*, *P. gingivalis, T. forsythia*, and *T. denticola*	Large study: DNA-DNA checkerboard hybridization	575, 576
	Periodontal infection	Review: Strong positive association between periodontal infection and prevalent hypertension	576
Myocardial infarction	Chronic dental infection correlated positively with MI	Association between dental chronic inflammatory diseases and the occurrence of acute myocardial infarction was studied	577– 579
	*Chlamydia pneumoniae,* *Helicobacter pylori*	Large study: 3315 case patients aged 75 years or younger	580
	Enterobacteria & influenza-like illness	Immunohistochemistry: Association study	582
Stroke (and TIA)	Comprehensive papers reviewing infection and stroke		585– 594
	Many bacterial species	84 different species detected in 77 patients	595, 596
	Community-acquired bacteremia	Population-based cohort study	597
	Bacterial endocarditis (Organisms found included *S. pneumonia*e, *N. meningitides* and other)	Culture of cerebrospinal fluid: Observational cross-sectional study	598
	*Borrelia burgdorferi*	ELISA	599
	*Brucella* spp.	*Brucella* agglutination and Coombs’ tests in blood	600
	*Chlamydia pneumoniae*	Serology	601– 603
	*Haemophilus influenzae*	Multivariate time series analysis to assess an association between infections and stroke using the established ‘3h-algorithm’	604
	*Mycobacterium tuberculosis*	Cox proportional hazard regressions	605
	*Mycoplasma pneumoniae*	Association between MP infection and risk of ischemic stroke; ELISA; serology	606– 608
	*Neisseria meningitidis*	Latex agglutination test and counterimmunoelectrophoresis	609
	*Staphylococcus aureus*	Prospective observational cohort study and retrospective review	610, 611
	*Streptococcus bovis*	Blood culture	612
	*Streptococcus mutans*	PCR	613
	*Streptococcus pneumonia*	Cox proportional hazard model	614
	*Streptococcus viridans*	Blood culture	615
	*Treponema pallidum*	Neurosyphillis also present Serology and *Treponema pallidum* haem agglutination test; rapid plasma regain test, and fluorescent treponemal antibody-absorption test *Serum and cerebrospinal fluid profiles for syphilis in Thai* *patients*	616, 617
	*Treponema pallidum*	Case study: Serology and haem agglutination test	616
Vascular disease (aneurysmal and lesions and atherosclerotic plaques)	Numerous bacterial species found in atheromas	Seven nonseptic patients: 6S rDNA analysis, biochemical tests, random amplification of polymorphic DNA PCR analysis, quantitative polymerase chain reaction (qPCR) and immunohistofluorescence	618
ENDOCRINE DISEASES			
**Diabetes**	Overview papers		624, 625
	Pseudomonads, *Stenotrophomonas maltophilia* and *Ps. aeruginoas*	PCR and antibodies from blood samples	626
type 1	*E. coli*, *Candida albicans,* enterovirus	Urine and blood culture: form patients with urinary tract infection	627– 629
	Various proteobacteria	PCR: 16SRNA form human blood	630
	Decreased bacteroidetes	Review paper	631
type 2	Systemic antibiotics improved diabetes control	Measured as a reduction in glycated hemoglobin or reduction in insulin requirements	632
	Many Gram-positives	qPCR: blood from patients	633
NEUROLOGICAL DISORDERS			
General	Comprehensive reviews		634– 636
Alzheimer’s Disease	Comprehensive reviews		637, 638
	*Porphyromonas gingivalis* *Chlamydia pneumoniae*	Immunolabeling and immunoblotting of brain tissue for the presence of LPS from *P. gingivalis* LPS will activate innate immune system in CNS and initiate pro- inflammatory cascades.	639
	Spirochetal bacteria	Comprehensive overview papers: Immunohistochemistry, Statistical correlation of a meta- analysis	640– 653
	*Helicobacter pylori*	Histology for diagnosis of Hp-I from AD patients	654– 656
		Population studies: eradication of bacteria versus state of dementia	655
		Animal (Rat) model	656
	*Actinomyces naeslundii*	Serum IgG levels in patients	657
Amyotrophic Lateral Sclerosis	*Mycoplasma* infections ( *M. fermentas, M. genitalium,* *M. penetrans, M. fermentans,* *M. hominis, M. pneumoniae*), *Chlamydia pneumoniae*, *Borrelia burgdorferi*	PCR, serology, microscopic observation: patient blood antibody analysis	436, 658– 660
Autism spectrum disorders	Mycoplasmal infections ( *M. fermentas, M. genitalium,* *M. penetrans, M. fermentans,* *M. hominis, M. pneumonia*)	PCR	661
	*Chlamydia pneumoniae* (co- infection with mycoplasma and human herpes virus-6), or wall-less bacteria	PCR: detected in blood of patients	663
		Critical review: amylotrophic lateral sclerosis (ALS)	662
Chronic depression	Numerous Gram-negatives from gut, e.g. *Hafnia alvei,* *Pseudomonas aeruginosa,* *Morganella morganii,* *Pseudomonas putida,* *Citrobacter koseri,* *Klebsiella pneumoniae*	IgA and IgM responses in patients	665
Parkinson’s Disease	*Helicobacter pylori*	^13^C urea breath test, odd ratios for the association between treatment for HP and risk of PD using logistic regression	666– 669
	*Toxoplasma gondii*	Serology, ELISA (IgG antibodies) patient-based study	670
	*Helicobacter suis*	DNA evidence: gastric biopsies of patients	671
Schizophrenia	*Toxoplasma gondii* (and Herpes simplex virus type 2)	A correlation between contact with house cats in early life and the development of schizophrenia exist	672– 676
	Prenatal exposure to bacterial infection in the first trimester increased risk of schizophrenia in the offspring	Prospective association study	677
	Toxoplasma, Mycoplasma and *Chlamydia trachomatis/* *pneumoniae*	Hypothesis paper	679
		Antibodies against bacteria in blood of patients	678, 679
OTHER INFLAMMATORY CONDITIONS			
Preeclampsia	*Tannerella forsythensis,* *Porphyromonas gingivalis,* *Actinobacillus* *actinomycetemcomitans,* *Prevotella intermedia,* *Fusobacterium nucleatum* *Treponema denticola* Significantly lowered risk following antibiotic treatment	PCR: placentas of 16 women	689
		Hypothesis and review	690
	Significant association with periodontal disease and UTI	Review papers	691– 694
	*Chlamydia pneumonia*	ELISA and qPCR of genomic DNA of bacteria from studies using many patients	695(but cf. 696)
	*Chlamydia trachomatis*	Serology: Antibodies were analyzed at a first prenatal visit (mean 14.2 weeks) and at delivery	697
	*Helicobacter pylori* *Chlamydia pneumonia*	Review paper discussing hypothesis of bacterial involvement in condition	698, 699
		Serology C-reactive protein (CRP), tumor necrosis factor alpha (TNFalpha), *Chlamydia pneumonia* IgG, IgM and plasma Helicobacter pylori IgA levels between 40 preeclamptic and 40 normal pregnant women	698
Chronic fatigue syndrome	Comprehensive reviews		701, 702
	*Hafnia alvei,* *Pseudomonas aeruginosa,* *Morganella morganii,* *Proteus mirabilis,* *Pseudomonas putida,* *Citrobacter koseri,* *Klebsiella pneumoniae*	Serum IgA and IgM against LPS Serology	700– 703
	Mycoplasmal infections ( *M. pneumonia, M. fermentans,* *M. honinis, M. penetrans*), *Chlamydia* pneumonia, Human herpes virus-6	PCR: *Conference proceedings*	704
	Various enterbacteria and others	IgG is patient blood	705
Vitamin D receptor (VDR) dysregulation	Cell wall deficient bacteria	Evade immune destruction by invading nucleated cells where they persist in the cytoplasm. From here they down-regulated the vitamin D receptor	706
	Multiple organisms, including mycrobacteria, *Borrelia*	Paper discusses a model describing how multiple species-bacterial, viral, and fungal-can cumulatively dysregulate expression by the VDR nuclear receptor	705
Antiphospholipid syndrome	*S. aureus*	A review paper: Cross-reacting antibodies	707
	Various viral and bacterial triggers	General review paper reviewing co-infections	708– 710
	*Toxoplasma*	Anti- *Toxoplasma* antibody screening in 98 patients with antiphospholipid syndrome	711
Sudden Infant Death Syndrome	*S. aureus*	Review papers: seasonality, bacteriology	712– 714
		Papers discuss markers of infection and inflammation are often found on autopsy along with microbial isolates	715, 716
		Toxaemic shock indicators in serum	717, 718
Other Inflammatory Bowel Diseases	Papers discussing dysbiosis of gut microbiota		719– 727
Sarcoidosis	*P. acnes*	*P. acnes* antibodies and antigens	728– 730
Migraine	*H. pylori*	A randomized, double blind, controlled trial	731, 732
		A meta-analysis of research between 2000 and 2013	732

## Relation between iron dysregulation, sepsis and other comorbidities

Many of the diseases in Table 3 are precisely those inflammatory diseases that we have listed before as coupled to iron dysregulation ^183, 184, 449, 452, 733^. A consequence of our analysis is that iron dysregulation and sepsis (as judged either by genuine infection by culturable bacteria or their inflammatory products such as LPS) should be associated causally with these various other diseases.

**Figure 9.**
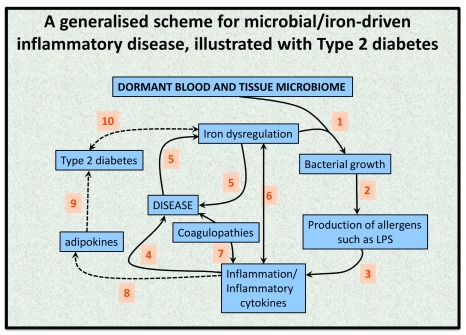
An elementary systems biology model of how iron dysregulation can stimulate dormant bacterial growth that can in turn lead to antigen production (e.g. of LPS) that can then trigger inflammation leading to cell death ^184^ and to a variety of diseases. While it is recognised that this simple diagram is very far from capturing the richness of these phenomena, there is abundant evidence for each of these steps, but sample references for the numbered interactions are (1) ^855– 858^ (especially including the release of free iron from ferritin ^452^), (2) ^859– 861^, (3) ^285, 473, 475, 862– 869^, (4) ^476, 733, 870– 873^, (5) ^183, 184, 452^, (6) ^874, 875^, (7) ^876– 882^, (8) ^883^, (9) ^884– 886^, (10) ^887, 888^.

This leads to a variety of predictions and postdictions that we rehearse. A purposely simple (and simplistic) indication of a plausible chain of events (for which each step is underpinned by substantial evidence) is given in Figure 9, both in general terms (for unspecified diseases) and for a couple of steps to type 2 diabetes. Figure 9 aims specifically to highlight the relationship between the ability of available iron to stimulate bacterial growth and the potential disease sequelae thereof.

### Iron and sepsis

First of all, it is well established that free iron may be raised in sepsis and related conditions ^734– 742^, as may serum ferritin ^743– 747^ (that has mainly dumped its iron ^452^). We have here argued that this is likely to be a significant contributor to the relationship between overt or cryptic infection and the many iron-related inflammatory diseases discussed here and elsewhere ^183, 184, 452, 733^. Note that patients suffering from iron overload diseases such as hereditary haemochromatosis are especially susceptible to infection (see e.g. 748– 750 and Table 3). Certainly the idea that iron-related metabolism and siderophores are virulence factors (e.g. 751– 763) is established unequivocally. In many diseases (e.g. lupus ^764, 765^ or type 1 diabetes ^766^) it is considered that patients with the disease are more prone to sepsis, but we suggest here that (as with stroke ^581, 585, 586, 588– 590, 767– 775^) it may more likely be the converse that is truer: patients suffering from latent infections are in fact more prone to acquiring, having, or exacerbating the state of these other conditions, in a vicious cycle (see Figure 9).

### Role of iron chelation in preventing sepsis

This was discussed at considerable length previously ^184^, and that discussion is not repeated here (though a few more recent and pertinent references include ^776– 779^). However, while (perhaps surprisingly, given what we see as the evidence) it does not even appear in the guidelines ^780^, there is considerable evidence ^184^ that appropriate iron chelation slows, inhibits or overcomes sepsis. We note, however, that some chelators are in fact known iron siderophores, and such molecules may assist the pathogen (e.g. 781– 783) and are to be avoided. On this basis, iron chelation may be a suitable alternative to antibiotics in preventing multiple inflammatory diseases (and such chelation may be nutritional rather than pharmacological in nature, e.g. 183). However, it is clear that we also need to learn to kill ‘dormant’ bacteria, and this usually requires that they are growing.

### Utility of antibiotics in treating non-communicable diseases

It is well established that the re-use of protein motifs in natural (and directed ^784^) evolution means that most drugs, especially the more lipophilic ones, are promiscuous in the sense that they bind to multiple targets ^194, 785^ (on average six known ones for marketed drugs ^786^). This said (and while we are very far from wishing to encourage the unnecessary use of antibiotics), the prediction here is that appropriate antibiotics will prove to have clinical benefit in diseases commonly seen as non-communicable. This is certainly known to be the case for a number of autoimmune diseases ^787^ such as rheumatoid arthritis ^788– 793^, multiple sclerosis ^794– 800^ and psoriasis ^801– 803^. Vaccination may prove equally effective ^804, 805^.

## Concluding comments: on the systems properties of dormancy and virulence

We have here brought together some of the relevant elements of environmental, laboratory, and clinical microbiology. We have argued that while their languages may differ (e.g. ‘dormancy’ vs ‘persistence’), very similar phenomena have been observed in each of these spheres (plausibly underlying a commonality of mechanism). Certainly the ability to culture microbes, and not merely to observe them (whether microscopically or via their macromolecular sequences or chemical products), remains an important goal of basic microbiology. This is likely to have significant payoffs in bioprospecting (e.g. 179, 806). However, we are sure that improved methods of detecting and identifying these dormant bacteria, whether this is done via chemical imaging, through macromolecular amplification and/or sequencing, or through resuscitation and culturing, will have a major role to play in increasing the awareness of their existence and importance.

Clearly dormant and/or persistent bacteria are likely to be relatively avirulent when they are in such dormant states, and able to bypass the attentions of the innate immune system (albeit the production of superantigens by at least some microorganisms ^807, 808^ may be what triggers autoimmune diseases). This ‘stealth’ antigenicity is probably why they have been largely unnoticed by us too ^809^, and their routine estimation via molecular methods ^810^ seems highly desirable. Indeed, virulence varies widely between individual strains (e.g. 811, 812). Modern molecular microbiology places much emphasis on the virulence of the pathogen, with concepts such as ‘pathogenicity islands’ ^813– 818^, ‘virulence genes’ ^819, 820^, and the ‘virulome’ ^821^ being commonplace. However, if dormant microbes resuscitate (or are to be resuscitated) *in vivo* we shall need to pay much more attention to the environmental triggers that can cause this to happen than we probably have so far ^822^ (given that the pathogen genotype is fixed ^823, 824^). In other words, virulence, like dormancy, is a phenotypic as well as a genotypic property. We remain largely ignorant of the means by which an optimal immune system has been selected for (or against) by longer-term evolution on the basis of microbial exposures in early life, and how this may have changed with more recent changes in human lifestyle ^825– 828^. Nor do we understand how such microbes might enter and exit blood cells (and see 62, 347, 829– 833) (albeit the known endosymbiotic origins ^834, 835^ of eukaryotic organelles must have presaged such mechanisms). Similarly, we do not yet know what may cause these dormant microbes to resuscitate (and/or to exit their intracellular niches). However, the potential for iron-associated replication and (e.g.) LPS production and shedding does provide a very straightforward explanation for the continuing low- or medium-grade inflammation characteristic of the many inflammatory diseases we have considered here and elsewhere ^183, 184, 449, 452, 733, 890^ ( Figure 9).

Recognising that correlation does not at all equate to causality (e.g. 195, 836), one approach to Science is based on varying independently something considered a cause and observing its predicted effects (e.g. 195, 837, 838). Temporal covariation of measurands can also be performed. The levels of free iron are clearly one such possibility. To assess causality in microbiology it is usual (e.g. 815, 839– 841) to invoke what are (variously ^842^) referred to the Henle-Koch or Koch’s postulates. These are based on the nature and presence, but not the physiological state, of an agent that might be believed to ‘cause’ (or at least contribute to) an infectious disease. Consequently, dormancy poses something of a challenge to the full completion of the required tests. Indeed a number of authors ^437, 815, 842– 845^ have recognised that these tests may need revision in the light of the ability to identify disease-causing microbes by sequence alone. We suspect that a key element here will be the ability to resuscitate dormant organisms *in vivo* and to see the effects of that on clinical disease.

From a ‘philosophy of science’ point of view (e.g. 841), one strategy taken to develop the evidence for a particular point of view hinges on the idea that if a series of ostensibly unrelated findings are brought together into a self-consistent narrative, that narrative is thereby strengthened. This is the strategy pursued here, and it is known as ‘coherence’ ^846– 848^.

As phrased by Silvers ^849^, “Several of our contributors showed how discoveries and insights could emerge with what seemed great promise, and yet be pushed aside, discarded, and forgotten – only to re-emerge once again, sometimes many years later, and become, in their new formulation, accepted as important”. In this sense, and as presaged in the opening quotation ^1^, it seems that ideas, as well as bacteria, can remain dormant for extended periods ^850, 851^.
