# Supplementary material for: Individuality, phenotypic differentiation, dormancy and ‘persistence’ in culturable bacterial systems: commonalities shared by environmental, laboratory, and clinical microbiology
Source: F1000Res. 2015 Sep 7;4:179. Originally published 2015 Jul 1. [Version 2] doi: 10.12688/f1000research.6709.2 (PMC4642849; doi:10.12688/f1000research.6709.2)
Supplement: Peer review history

# Peer review: Referee response for version 2

**DOI:** 10.5256/f1000research.7493.r11111

**Published:** 2015-11-06

**Authors:** Gerald Domingue

**Affiliations:** 1 Professor Emeritus, Tulane University, New Orleans, LA, USA

This review paper is an important modern perspective on bacterial persistence and expression of disease. The role of ‘stressed’, atypical, cell wall-defective, cryptic, pleomorphic forms in chronic inflammatory diseases in clinical medicine and especially in diagnostic pathology and clinical microbiology is grossly neglected and overlooked.  This paper warrants publication for its timely approach to a vastly important overlooked topic in science and medicine and its relevant, useful 890 cited references.  I see no point in further elaboration on semantics: persistence vs. dormancy.  In my opinion, regardless of terminology preferred (or debated) the relevant and important fact is identity of atypical forms in tissues, their basic biology and their relationship to disease.  

Although there is provocative circumstantial evidence linking pleomorphic forms suspected of being bacterial in origin in a wide array of chronic diseases, many were categorized as autoimmune, unrelated to microbes (unambiguous proof lacking). While the persistence of stainable forms (various structures) are often seen in tissue specimens utilizing histopathologic and bacteriologic stains, most are discarded as insignificant staining artifacts and debris, and especially in the absence of non-cultivable bacteria from accompanying specimens.  In my opinion, this is a primary reason the significance of such stainable findings has been ignored in clinical medicine and has stymied their identity as causative agents of disease. Furthermore, even when there may be growth of atypical bacterial forms on artificial culture media, bizarre (non-standard) morphologic, biochemical, physiological characteristics of the isolated organism *in vitro*, the findings are most often disregarded as “contaminants”. 

Permit me to digress and cite such an example of an unidentified pleomorphic form isolated from patients with interstitial cystitis (a chronic, debilitating disease of unknown origin).  These atypical isolates were subjected to elaborate, microbiological, immunological, biochemical, physiologic and electron microscopic characterizations. The findings (data) were presented at the American Urological Association annual meeting, only to have a well-known academic urologist and interstitial cystitis specialist congratulate the researchers for the elaborate experimental description of an ‘artifact’.  Obviously with that type of unexpected and disappointing comment, there was nothing more to be said, other than, thank you! So there you have it:  The regrettable dismissal of a potentially important finding in a disease of unknown origin by an individual who could not see the forest through the trees and without evidence to substantiate the claim that the finding was an ‘artifact’. 

The 14 topics and subsections outlined in Figure 4 of the review set the stage for the “dramatics” – ‘mind map’ – that follows. In itself this graphic may have been enough when accompanied by germane references instead of lengthy written discussions for each topic since much is *déjà vu*, gleaned over a period of many decades from published findings.  On the other hand, it is often useful to repeat, for emphasis, and especially to call attention to neglected topics, which I suspect was the intent.  Although the tables and graphics are worthwhile, it does take time to digest it all, meaning it may have been possible to shorten the paper.

Two important publications (not cited in list of 890 references): companion papers by Green *et al *in Infection and Immunity, 1974, Oct; 10 (4): 889-914 and 915-927; demonstrated the phenomena of microbial persistence and reversion with *Streptococcus faecalis *L-forms in human embryonic kidney cells, followed by a proposed reproductive cycle for a relatively stable L-phase variant of *Streptococcus faecalis*.  I call these publications to the attention of the authors because of their possible application to the fundamental basis of persistence by ‘stressed’, atypical bacteria in chronically diseased human subjects.  Essential to the thesis of Green *et al *is that small, electron dense, non-vesiculated L-forms were shown to be the central (core) element in bacterial persistence in these experimental studies. The researchers concluded that depending on the stimulus received, these dense forms might be considered as undifferentiated cells, with the capacity to develop along several different routes. *In vitro*, the dense form was observed to divide and bud rapidly. In addition, the dense forms appeared to be capable of growth and development within vesicles of mature mother forms.  When these forms were released from the vesicles into the surrounding fluid medium, further growth occurred, resulting in the development of immature and ultimately mature mother forms. Under conditions unfavorable for L-form growth, these dense forms developed first into transitional forms and then into the bacterial form. These dense forms might therefore be considered as undifferentiated ‘stem cells’ with the capacity to develop along several different routes, depending upon the stimulus received.  Hence, in applying these findings to altered forms created *in vivo* (humans) these may take up intracellular and/or extracellular residence; possibly establishing a sort of immune protected parasitic relationship persisting/surviving phagocytic action, and creating subtle pathologic changes in the host during a prolonged period of tissue persistence.  This might translate into an etiology for chronic inflammatory diseases, when the ‘stressed’ bacteria increase in numbers and overwhelm the normal biological functions of the host.  I further propose that *in vivo* persistence of these bacterial elements escape immune surveillance partially, completely, or may integrate with host cell organelles to create bacteria-host cell-antigen complexes which could provoke immunopathologic consequences. Highly relevant, recently published data on modifications of gene expression, modes of division for stressed bacteria, and the paradoxical finding of peptidoglycan in L-forms are pertinent to the hypothesis that atypical, pleomorphic bacteria are the organisms responsible for persistence and expression of disease. Finally, it is hoped that the Kell, Potgieter, Pretorius timely, interesting and provocative review will call attention to this highly significant, too often overlooked subject.  In my opinion, this review calls for a scientific/medical challenge: 1) to motivate visionary scientists and clinicians to investigate the fundamental origins of bacterial persistence in chronic diseases; 2) to unambiguously identify tissue persisting forms utilizing modern molecular technology, and 3) to design elegant experiments to provide convincing scientific proof (or disprove) that extracellular and or intracellular stainable bodies observed in histopathologic specimens and dense bodies at the electron microscopic level (culture negative) are bacteria existing as ‘stressed’ altered forms in tissues and not tissue or staining artifacts.  Proof of the above hypothesis would open new arenas in clinical diagnosis, management and treatment of numerous chronic inflammatory human diseases of unknown etiology and might even extend to a bacterial cause for certain malignancies (as previously proposed many decades ago).

I have read this submission. I believe that I have an appropriate level of expertise to confirm that it is of an acceptable scientific standard.

# Peer review: Referee response for version 1

**DOI:** 10.5256/f1000research.7206.r9285

**Published:** 2015-08-11

**Authors:** Vanya Gant

**Affiliations:** 1 Department of Medical Microbiology, University College London Hospitals NHS Foundation Trust, London, UK

I review Kell *et al’s *review relating to individuality, phenotypic differentiation, dormancy and “persistence” as a clinical microbiologist, infectious diseases doctor, with an interest in developing and assessing the impact of rapid sequence-based molecular blood and lung diagnostics in the critically ill.

 This review reminded me of Mussorsky’s *Pictures at an Exhibition,* a collection of hastily composed pieces whose theme was to take an interested individual through an art gallery, and to tarry awhile in front of 10 *Tableaux*, interspersed with musical elements referring to the “Promenade” through the gallery.

And so it is with Kell *et al’s* review. After an introductory *Promenade *relating to matters of bacterial dormancy and its relationship with just about any other conceivable physical state between life and death, exhaustively referenced together with the thought provoking *Postgate-*ian concept of the difficulties inherent in differentiating bacterial life from death if you only have an instant in time to measure it – we are then presented with several pictures, garlanded for us in extensively referenced detail by the authors. Were mindmaps not enough to capture the reader’s curiosity as to this *magnum opus* of a kind, we are invited to walk through Kell *et al’s* gallery of mental pictures depicting scenes of the Yet to be Cultured, Those bacteria that aren’t culturable yet but are certainly not dead, the biological importance of bacterial pheromones, the evils of Iron - thence to the Clinical Microbiology Room of Pictures with a liberal helping of systems biology throughout.

I am a proponent of, and believer in, the present and future potential of Nucleic Acid Technology (NAT) for pathogen detection in Clinical microbiology and I use such techniques on a daily basis. When appropriately deployed, it allows me to find those “unculturable” pathogens as drivers for individual clinical cases of infection. Perhaps strangely, this is a relatively new paradigm for most practising clinicians, and one which likely will generate fundamental discoveries highly relevant to human disease, and for all we know as equally important as *Helicobacter. *That such sequences should be found in blood is hardly surprising, given that human beings have between 10 and 100 times more bacterial cells than their own, living (or persisting, or dormant) on and in them. This groups’ demonstration of bacteria adhering to red cells (also *in* red cells) is certainly very intriguing, and such suggested “atopobiosis” is more expansively dealt with in another publication and prompts far more questions than it answers – in a good way. Another obvious question relates to how these adherent bacteria may remain undetected and intact in the presence of numerous moieties central to both innate and acquired immunity (complement and antibody to name but two) as well as escaping phagocytosis in the liver and spleen. It would certainly be interesting to look at red cells in the grave condition of erythrophagocytosis, a condition whose mechanism is in most cases obscure –it might even be that adherent bacteria “opsonize” the red cells in these cases. This reader, however, does baulk at the very serious work to be done as regards untangling the mechanistic nature of an “association” with several diseases, and certainly at this stage it would be very unwise to suggest it’s anything more than that. Further work of this nature should be approached and undertaken with extreme caution and rigor in view of the myriad possible explanations other than causative ones; the Measles vaccine/autism saga comes to mind here.

It is likely therefore that such technologies will perforce “lift the lid” on what might lie beyond the Culturable, and its relationship to human disease. This is explored in Table 3, which represents a *tour de force* as concerns the sheer volume of references relating to all that appears to associate human Disease and organisms, mostly bacteria.

Unfortunately, this Table doesn’t work for me. Whilst it will serve me as a unique and accessible resource of information in this space, it is anarchic. Correctly described as “Evidence for agents in non-communicable diseases”, it lists, in no particular order, and with no apparently critical eye, references 470 to 712 as relevant to the Table subject stated above. This list’s breadth as concerns both organisms and clinical diseases is extraordinary; and the literature quoted in a table described as “effect of bacterial involvement” ranges from unusual cases, to mechanistic assumptions of what LPS might do, to the concept of “dysbiosis” amongst many others. I was left rather dizzy from the mental exercise needed to constantly adjust to the sheer scale and variation of why a particular organism, or something it produces, might either directly causally relate to a particular disease, or perhaps through the individuals’ immune response to it; especially now we know how outbred we are as concerns immune responsiveness.

This review finishes with an impressive and lyrical chiding for Scientists, whereby those who research this field should wake up from their intellectual slumber, as might and indeed do bacteria.

This review is additionally peppered with tantalizing if perhaps sometimes unfounded assumptions, some arguable and some bordering on plain unreasonable. Certainly my eyebrow raising went into overdrive when considering Kell’s conviction as concerns a Catholic Grand Unifying Theory based around the Evils of Iron, the subject of a previous equally grand *Magnum Opus*.

This review has to be one of the most undisciplined I have read in a long time, on occasions associating seemingly disparate observations  and conflating “scientifically” determined facts with clinical issues.

Having said this, I should finish by applauding Kell *et al’s* review as a thumping good read. It’s fast paced, edgy, a real treasure trove of papers for me to read at leisure, and goes way outside the usual, expected and conventional  boundaries of style of prose and rigor we “normally expect” of such scientific publications.  And (warts and all, and there are many) it left this reader thinking that there indeed is Life beyond dormancy within the review’s style itself,  beyond the doubtless very important but less imaginative run-of-the-mill, tightly written yet dreary “Scientific Publication”. It is almost as if this review in all its unconventionality were particularly well aligned to the current state of the Art for the Uncultured in Clinical Medicine (bacteria, not Doctors) and its potential to release significant Paradigm shifts.  No doubt this reviews’ readers are made up of those who have the capacity to appreciate Kells’ latest brand of emergent, imaginative systems biology style of thinking underneath what some might consider a publication of inadequate scientific rigor.

I have read this submission. I believe that I have an appropriate level of expertise to confirm that it is of an acceptable scientific standard.

# Response

**Published:** 2015-08-18

**Authors:** Douglas Kell

**Affiliations:** The University of Manchester, UK

- *"I review Kell et al’s review relating to individuality, phenotypic differentiation, dormancy and “persistence” as a clinical microbiologist, infectious diseases doctor, with an interest in developing and assessing the impact of rapid sequence-based molecular blood and lung diagnostics in the critically ill.*
*This review reminded me of Mussorsky’s Pictures at an Exhibition, a collection of hastily composed pieces whose theme was to take an interested individual through an art gallery, and to tarry awhile in front of 10 Tableaux, interspersed with musical elements referring to the “Promenade” through the gallery.*
*And so it is with Kell et al’s review. After an introductory Promenade relating to matters of bacterial dormancy and its relationship with just about any other conceivable physical state between life and death, exhaustively referenced together with the thought provoking Postgate-ian concept of the difficulties inherent in differentiating bacterial life from death if you only have an instant in time to measure it – we are then presented with several pictures, garlanded for us in extensively referenced detail by the authors. Were mindmaps not enough to capture the reader’s curiosity as to this magnum opus of a kind, we are invited to walk through Kell et al’s gallery of mental pictures depicting scenes of the Yet to be Cultured, Those bacteria that aren’t culturable yet but are certainly not dead, the biological importance of bacterial pheromones, the evils of Iron - thence to the Clinical Microbiology Room of Pictures with a liberal helping of systems biology throughout."*
  This is a lovely analogy, which we shall let readers enjoy in the open referee’s report; we are probably not capable of recasting the review in Mussorgskian style anyway! In this regard, readers might also enjoy a little known and whimsical piece on bioinformatics that takes just such an approach: Goble C, Wroe C: The Montagues and the Capulets. Comp Func Genomics 2004; 5:623-632.
- *"I am a proponent of, and believer in, the present and future potential of Nucleic Acid Technology (NAT) for pathogen detection in Clinical microbiology and I use such techniques on a daily basis. When appropriately deployed, it allows me to find those “unculturable” pathogens as drivers for individual clinical cases of infection. Perhaps strangely, this is a relatively new paradigm for most practising clinicians, and one which likely will generate fundamental discoveries highly relevant to human disease, and for all we know as equally important as Helicobacter. That such sequences should be found in blood is hardly surprising, given that human beings have between 10 and 100 times more bacterial cells than their own, living (or persisting, or dormant) on and in them. This groups’ demonstration of bacteria adhering to red cells (also in red cells) is certainly very intriguing, and such suggested “atopobiosis” is more expansively dealt with in another publication and prompts far more questions than it answers – in a good way. Another obvious question relates to how these adherent bacteria may remain undetected and intact in the presence of numerous moieties central to both innate and acquired immunity (complement and antibody to name but two) as well as escaping phagocytosis in the liver and spleen. It would certainly be interesting to look at red cells in the grave condition of erythrophagocytosis, a condition whose mechanism is in most cases obscure –it might even be that adherent bacteria “opsonize” the red cells in these cases. This reader, however, does baulk at the very serious work to be done as regards untangling the mechanistic nature of an “association” with several diseases, and certainly at this stage it would be very unwise to suggest it’s anything more than that. Further work of this nature should be approached and undertaken with extreme caution and rigor in view of the myriad possible explanations other than causative ones; the Measles vaccine/autism saga comes to mind here."*
  These are excellent points, and we have covered some of them in the forward-looking concluding section. While they might be seen as ‘premature’ (in the sense that it requires acceptance of the basic ‘dormancy’ hypothesis in the first place) they do point to important areas where we would seek a mechanistic understanding of what is going on.
- *"It is likely therefore that such technologies will perforce “lift the lid” on what might lie beyond the Culturable, and its relationship to human disease. This is explored in Table 3, which represents a tour de force as concerns the sheer volume of references relating to all that appears to associate human Disease and organisms, mostly bacteria.*
*Unfortunately, this Table doesn’t work for me. Whilst it will serve me as a unique and accessible resource of information in this space, it is anarchic. Correctly described as “Evidence for agents in non-communicable diseases”, it lists, in no particular order, and with no apparently critical eye, references 470 to 712 as relevant to the Table subject stated above. This list’s breadth as concerns both organisms and clinical diseases is extraordinary; and the literature quoted in a table described as “effect of bacterial involvement” ranges from unusual cases, to mechanistic assumptions of what LPS might do, to the concept of “dysbiosis” amongst many others. I was left rather dizzy from the mental exercise needed to constantly adjust to the sheer scale and variation of why a particular organism, or something it produces, might either directly causally relate to a particular disease, or perhaps through the individuals’ immune response to it; especially now we know how outbred we are as concerns immune responsiveness."*
  We very much accept the point that the table could be improved with regard to ordering, and we have done so accordingly. However, we think that readers will recognise it for what it is (as does the referee), viz. as a useful resource and/or pointer to a large literature in which specialists in disease X may wish to read at least those papers we suggest as relevant to ‘their’ disease, while others will simply see it as a recognition of the widespread evidence for our more general claims.
- *"This review finishes with an impressive and lyrical chiding for Scientists, whereby those who research this field should wake up from their intellectual slumber, as might and indeed do bacteria.*
*This review is additionally peppered with tantalizing if perhaps sometimes unfounded assumptions, some arguable and some bordering on plain unreasonable. Certainly my eyebrow raising went into overdrive when considering Kell’s conviction as concerns a Catholic Grand Unifying Theory based around the Evils of Iron, the subject of a previous equally grand Magnum Opus."*
  As mentioned in the comments on the review of referee 1, the basis for this is the desire to produce a coherent story (in the sense used by Philosophers of Science), and (as referee 1 also states) it is well known that microbial growth in vivo is normally limited by iron availability. That iron dysregulation is also a hallmark of **just** those chronic inflammatory diseases that we highlight here is consistent with this view, and indeed serves to provide a simple explanation for this. Of course, as the referee indicates (and referee 1 does too), further demonstrations will benefit from varying iron levels as an independent variable.
- *"This review has to be one of the most undisciplined I have read in a long time, on occasions associating seemingly disparate observations  and conflating “scientifically” determined facts with clinical issues.*
*Having said this, I should finish by applauding Kell et al’s review as a thumping good read. It’s fast paced, edgy, a real treasure trove of papers for me to read at leisure, and goes way outside the usual, expected and conventional  boundaries of style of prose and rigor we “normally expect” of such scientific publications.  And (warts and all, and there are many) it left this reader thinking that there indeed is Life beyond dormancy within the review’s style itself,  beyond the doubtless very important but less imaginative run-of-the-mill, tightly written yet dreary “Scientific Publication”. It is almost as if this review in all its unconventionality were particularly well aligned to the current state of the Art for the Uncultured in Clinical Medicine (bacteria, not Doctors) and its potential to release significant Paradigm shifts.  No doubt this reviews’ readers are made up of those who have the capacity to appreciate Kells’ latest brand of emergent, imaginative systems biology style of thinking underneath what some might consider a publication of inadequate scientific rigor."*
  Many thanks for these last comments; we have nothing further to add here.

# Peer review: Referee response for version 1

**DOI:** 10.5256/f1000research.7206.r9602

**Published:** 2015-07-23

**Authors:** Michael Barer

**Affiliations:** 1 Department of Infection, Immunity and Inflammation, University of Leicester, Leicester, UK

Kell, Potgieter and Pretorius present a stimulating and argumentative review ranging from the interrelationships between the culturablilty of bacteria and their viability and any links these descriptions may have to defined physiological states, through a discussion of environmental bacteria and ultimately focusing on the human-associated microbiota, particularly those found in blood (without associated symptoms of sepsis) and their proposed roles in disease. Two central themes are developed beyond those that have been discussed extensively elsewhere: 1) the proposal that failure to culture bacteria from many samples often reflects dormancy and 2) that such dormant bacteria interact with host iron regulation to contribute to or directly cause a panoply of chronic diseases largely labelled as non-communicable.

At a general level I support the provocative stance taken by the authors.  With 861 cited references, at the very least they provide a valuable resource for anyone wishing to consider the potential microbial contribution to diseases traditionally considered free of this aetiological component. Of course *Helicobacter *infection stands as a monument to the stupidity of dismissing this possibility in the face of carefully assembled evidence. Indeed this reviewer, who many years ago, was presented with a case of duodenal ulcer in his final medical exams, would probably have experienced quite a different career had he claimed a role for infection in causing his patient’s pathology.

In considering the specific points presented I have multiple concerns, the most significant of which I will indulge in outlining below.

Semantics present a central problem in considering bacterial viability and physiology and I broadly support the approach taken here. The authors do try to define their terms but some problems remain. In particular I take issue with the very broad application of term “Persisters” which should be reserved for cells that survive (have the potential to replicate) after exposure to an antimicrobial stress to which kills most cells in an actively growing culture of the organism concerned. Conflation of this term with “Dormancy” implies on the one hand that the persisting cells must have been dormant and on the other that dormancy and persistence represent the same physiological state in bacteria. This difficulty resurfaces later when they define dormancy but other problems emerge before then.

I was next concerned by the extensive use of the term “Differentiation”.  I completely agree that what we used to think of as uniform bacterial populations are probably never so but the degree to which subpopulations may be considered differentiated rather than reflecting a range of adaptive responses or indeed, some degree of injury, is not considered here and again I think this leads to problems in considering their hypotheses under a unitary banner downstream. I consider differentiation to require phenotypic changes that are not directly reversible, as in the case of sporulation, whereas adaptation can involve expression of a single gene that can be reversed by its subsequent repression.  I do agree that cell cycle contributes to the range of phenotypes in a pure bacterial culture and that this is not the only reason for their diversity (but was not enlightened by use of the term “ *modulo*” in this regard).

The operational definition of dormancy given deliberately leaves open the possibility of metabolic activity and seems only to require that the cell so defined should not divide; this did not allow me to recognise which operational tests might be applied to enumerate or detect dormant cells. Subsequently the detection of molecular signals indicative of bacterial presence in samples from which they were not isolated in culture is taken as evidence of dormancy. In the first case do we accept any non-dividing cell as dormant and in the second I can (and will) offer multiple alternate explanations other than dormancy. Moreover, returning briefly to the conflation between dormancy and persisters, the recent work of John McKinney and colleagues shows that antibiotic exposed persisting cells are not necessarily non-dividing cells in the mycobacterial system he studied.

Alternative interpretations of the presence of bacterial 16SrDNA sequences in blood when culture fails to detect the organisms from which they derive, include the presence of dead, injured or moribund cells. If they are shown to be repeatedly present then they must either be able to persist in the face of clearance mechanisms or be supplied at a rate equal to their clearance; both seem equally plausible to the dormancy explanation to me. Moreover, why the first three explanations offered for “Not-yet-cultured” should apply to environmental bacteriology but not to clinical samples escapes me.

I am led to the conclusion that the authors have chosen to label evidence for discrepancies between culture and nucleic acid detection of bacteria in blood to give their hypotheses a simple headline. I have no problem with the proposal that human blood and tissues classically considered sterile in the absence of overt symptoms of infection are frequently exposed to bacteria and bacterial products that in many cases contribute to serious chronic disease. However, I consider the burden of available evidence currently provides many potential explanations within the field of microbiomics/metagenomics in contrast to the dormancy hypothesis offered here. Further, I feel this broad application of dormancy to bacterial phenotypes which, even in the case of Rpf dependency, have not been shown to result from a programme of gene expression that could be considered as differentiation, diminishes the value of the term. Indeed there remains no direct proof that dormancy of *Mycobacterium tuberculosis* underpins what we call latent tuberculosis infection and it is not essential to the observed clinical or pathological pattern, notwithstanding the widespread acceptance of this view by most researches, including me.

I am not fundamentally opposed to the ideas presented by Kell and colleagues but I do not think they are assisted by lack of attention to the contradictions I have identified above.

Finally I come to the iron dysregulation hypothesis and its pro-inflammatory consequences. It is beyond my expertise to comment on the plausibility of the inorganic chemistry deployed here or to review the evidence relating to more than a fraction of the conditions listed. The importance of the struggle between pathogens and host for access to iron is beyond question. When I entered the medical field of infectious disease it was fully recognised that depriving bacteria from iron was a potential therapeutic angle and indeed iron chelation was studied. Desferioxamine, a widely used agent in iron overload, was investigated and found to effectively deliver iron to the pathogen and the approach was set aside. More recently this agent has been identified as a major risk factor in serious fungal infection and guidance specifically recommends its avoidance.   Newer agents seem not to suffer from this problem and the approach deserves renewed attention. However, I would not underestimate the ability of pathogens to outwit our pharmaceutical industry in the battle to sequester iron. While there are reasons beyond the host –pathogen tug-of war for iron to consider chelation as a therapeutic option, the potential for adverse effects is significant and I think the suggestion that omission of iron chelation from recent guidance on sepsis management is “shocking” is not justified.

Focussing briefly on the specific diseases cited and their relation to bacterial exposure in one form or another, I find that evidence cited frequently rests on what can be considered “fringe” hypotheses that have little currency in their respective fields. This is not to discourage their continued pursuit but it does weaken the strength of the authors’ argument when investigation of the supporting literature frequently leads to papers that are given little credence in the specialist field.  Of course “cave *Helicobacter”* must remain on the table. But there, an accidental technical breakthrough led to an avalanche of convincing laboratory and clinical data.

In summary Kell, Potgieter and Pretorius have produced an interesting read which bring many important ideas to our attention. I am not convinced of the breadth of conditions to which they argue their ideas are applicable and I await with interest, demonstration of of how they may be practically pursued and some selected definitive proofs that iron-driven inflammatory disease is as important as they claim.

I have read this submission. I believe that I have an appropriate level of expertise to confirm that it is of an acceptable scientific standard, however I have significant reservations, as outlined above.

# Response

**Published:** 2015-08-18

**Authors:** Douglas Kell

**Affiliations:** The University of Manchester, UK

- *"Kell, Potgieter and Pretorius present a stimulating and argumentative review ranging from the interrelationships between the culturablilty of bacteria and their viability and any links these descriptions may have to defined physiological states, through a discussion of environmental bacteria and ultimately focusing on the human-associated microbiota, particularly those found in blood (without associated symptoms of sepsis) and their proposed roles in disease. Two central themes are developed beyond those that have been discussed extensively elsewhere: 1) the proposal that failure to culture bacteria from many samples often reflects dormancy and 2) that such dormant bacteria interact with host iron regulation to contribute to or directly cause a panoply of chronic diseases largely labelled as non-communicable.*
*At a general level I support the provocative stance taken by the authors.  With 861 cited references, at the very least they provide a valuable resource for anyone wishing to consider the potential microbial contribution to diseases traditionally considered free of this aetiological component. Of course Helicobacter infection stands as a monument to the stupidity of dismissing this possibility in the face of carefully assembled evidence. Indeed this reviewer, who many years ago, was presented with a case of duodenal ulcer in his final medical exams, would probably have experienced quite a different career had he claimed a role for infection in causing his patient’s pathology.*
*In considering the specific points presented I have multiple concerns, the most significant of which I will indulge in outlining below."*
  Many thanks for the above; it is perfectly accurate and we have nothing to add here.
- *"Semantics present a central problem in considering bacterial viability and physiology and I broadly support the approach taken here. The authors do try to define their terms but some problems remain. In particular I take issue with the very broad application of term “Persisters” which should be reserved for cells that survive (have the potential to replicate) after exposure to an antimicrobial stress to which kills most cells in an actively growing culture of the organism concerned. Conflation of this term with “Dormancy” implies on the one hand that the persisting cells must have been dormant and on the other that dormancy and persistence represent the same physiological state in bacteria. This difficulty resurfaces later when they define dormancy but other problems emerge before then."*
  This is entirely fair; we see that we occasionally elided the terms ‘dormancy’ and ‘persistence’ to imply synonymy, when either there is none or at least there is no evidence for it. We think the best solution is to add a little section pointing out the semantic difficulties, repeating the operational nature of the definitions, and specifying that in very few cases do we actually know the true physiological state of individual cells – which is what matters with regard to replicatory potential. This material mainly appears in the section defining dormancy, and its title has been extended to note the semantic issues.
- *"I was next concerned by the extensive use of the term “Differentiation”.  I completely agree that what we used to think of as uniform bacterial populations are probably never so but the degree to which subpopulations may be considered differentiated rather than reflecting a range of adaptive responses or indeed, some degree of injury, is not considered here and again I think this leads to problems in considering their hypotheses under a unitary banner downstream. I consider differentiation to require phenotypic changes that are not directly reversible, as in the case of sporulation, whereas adaptation can involve expression of a single gene that can be reversed by its subsequent repression.  I do agree that cell cycle contributes to the range of phenotypes in a pure bacterial culture and that this is not the only reason for their diversity (but was not enlightened by use of the term “modulo” in this regard)."*
  We mainly agree, and suggest what we think is a useful clarification or extension. We note again that “reversibility” is established post hoc, but there are at least two meanings involved. At one level we are discussing a reversibility of states. Let us take a spore and a vegetative cell, which obviously, for sporulating bacteria, can indeed interconvert (“reversibly”). However, another level or meaning implies a mechanistic reversibility, i.e. the path from A to B is simply traversed in the opposite direction when B reverts or interconverts to A. Not only is this not what we mean but (also for thermodynamic reasons) it is certainly not what is done (sporulation and germination in *B. subtilis* are definitely quite separate processes, as indicated by the referee, and one is not at all the reverse of the other). We have added clarificatory comments accordingly. (One might also have added, but we have not in the ms as it would distract, that similar issues apply to the ‘reversibility’ of enzymes and of biochemical pathways (gluconeogenesis is not mechanistically a reversal of glycolysis, even if the “start” and “end” states are the same molecules.)
- *"The operational definition of dormancy given deliberately leaves open the possibility of metabolic activity and seems only to require that the cell so defined should not divide; this did not allow me to recognise which operational tests might be applied to enumerate or detect dormant cells. Subsequently the detection of molecular signals indicative of bacterial presence in samples from which they were not isolated in culture is taken as evidence of dormancy. In the first case do we accept any non-dividing cell as dormant and in the second I can (and will) offer multiple alternate explanations other than dormancy. Moreover, returning briefly to the conflation between dormancy and persisters, the recent work of John McKinney and colleagues shows that antibiotic exposed persisting cells are not necessarily non-dividing cells in the mycobacterial system he studied."*
  The hallmark of the dormant macrostate, stated in quotation marks in the second paragraph of the ‘dormancy’ section, is indeed that the cells in question do not immediately grow when attempts to culture them under “suitable” conditions (that normally admit their growth), are often (but not necessarily) of low metabolic activity, but are not operationally dead since they can be resuscitated. On this basis we think that this should allow the referee or anyone else to determine the operational tests. It follows that we do not accept ‘any’ non-diving cell as dormant since only resuscitable cells can – *post hoc* – be considered dormant, and certainly a non-dividing cell it may be irreversibly injured or operationally dead. However, the presence of molecular signals (e.g. 16S) in samples from which nothing (or many fewer colonies or OTUs) may be recovered by culture is certainly an indication of the possibility of resuscitation, and hence dormancy.
  The referee is entirely correct that we had missed John McKinney’s recent and very relevant work, and we mention it accordingly.
- *"Alternative interpretations of the presence of bacterial 16SrDNA sequences in blood when culture fails to detect the organisms from which they derive, include the presence of dead, injured or moribund cells. If they are shown to be repeatedly present then they must either be able to persist in the face of clearance mechanisms or be supplied at a rate equal to their clearance; both seem equally plausible to the dormancy explanation to me. Moreover, why the first three explanations offered for “Not-yet-cultured” should apply to environmental bacteriology but not to clinical samples escapes me."*
  The referee is entirely correct with regard to the last sentence, and the whole point (or at least a major theme) of our review is precisely that what is well established in environmental microbiology has had much less impact in clinical microbiology (referee 2 makes this exact point, even more explicitly). We agree that in a steady state such cells must be supplied at a rate equal to that of their clearance, and that the fact that clearance is lower than probably expected implies a significant ability to evade the innate and adaptive immune systems. We also take it that for common organisms (not very slow growers such as certain mycobacteria) the former rates must be much lower than those typically attainable in laboratory cultures, else we would have classical sepsis. We have added a few comments on these issues accordingly, in the section entitled ‘Generalised failure of classical techniques to detect dormant bacteria in clinical microbiology’.
- *"I am led to the conclusion that the authors have chosen to label evidence for discrepancies between culture and nucleic acid detection of bacteria in blood to give their hypotheses a simple headline. I have no problem with the proposal that human blood and tissues classically considered sterile in the absence of overt symptoms of infection are frequently exposed to bacteria and bacterial products that in many cases contribute to serious chronic disease. However, I consider the burden of available evidence currently provides many potential explanations within the field of microbiomics/metagenomics in contrast to the dormancy hypothesis offered here. Further, I feel this broad application of dormancy to bacterial phenotypes which, even in the case of Rpf dependency, have not been shown to result from a programme of gene expression that could be considered as differentiation, diminishes the value of the term. Indeed there remains no direct proof that dormancy of Mycobacterium tuberculosis underpins what we call latent tuberculosis infection and it is not essential to the observed clinical or pathological pattern, notwithstanding the widespread acceptance of this view by most researches, including me.*
*I am not fundamentally opposed to the ideas presented by Kell and colleagues but I do not think they are assisted by lack of attention to the contradictions I have identified above."*
  All of the above is entirely fair, and we do not disagree. We hope that the changes we have now made to the ms to weaken the ostensible claims (and misplaced synonymies) now meet the referee’s approval. For instance we have stressed that while the presence of suitable molecular sequences (e.g. 16S) implies that it is worth seeking to resuscitate the organisms from which it came, an absence would imply that it is not. A success in resuscitating organisms from a sample that initially appeared sterile would from our operational definition imply that those ones were indeed dormant, and we’d like to think that this had now been clarified.
- *"Finally I come to the iron dysregulation hypothesis and its pro-inflammatory consequences. It is beyond my expertise to comment on the plausibility of the inorganic chemistry deployed here or to review the evidence relating to more than a fraction of the conditions listed. The importance of the struggle between pathogens and host for access to iron is beyond question. When I entered the medical field of infectious disease it was fully recognised that depriving bacteria from iron was a potential therapeutic angle and indeed iron chelation was studied. Desferioxamine, a widely used agent in iron overload, was investigated and found to effectively deliver iron to the pathogen and the approach was set aside. More recently this agent has been identified as a major risk factor in serious fungal infection and guidance specifically recommends its avoidance.   Newer agents seem not to suffer from this problem and the approach deserves renewed attention. However, I would not underestimate the ability of pathogens to outwit our pharmaceutical industry in the battle to sequester iron. While there are reasons beyond the host –pathogen tug-of war for iron to consider chelation as a therapeutic option, the potential for adverse effects is significant and I think the suggestion that omission of iron chelation from recent guidance on sepsis management is “shocking” is not justified."*
  The point about desferrioxamine is well made (and we mention it, with citations), but the molecule is of course in fact a natural prokaryotic siderophore, from *Streptomyces pilosus.* We have replaced the term ‘shocking’ with something more suitable.
- *"Focussing briefly on the specific diseases cited and their relation to bacterial exposure in one form or another, I find that evidence cited frequently rests on what can be considered “fringe” hypotheses that have little currency in their respective fields. This is not to discourage their continued pursuit but it does weaken the strength of the authors’ argument when investigation of the supporting literature frequently leads to papers that are given little credence in the specialist field.  Of course “cave Helicobacter” must remain on the table. But there, an accidental technical breakthrough led to an avalanche of convincing laboratory and clinical data."*
  It is probably a philosophical distraction to rehearse how often in science something outside the mainstream is blocked for many years by ‘vested interests’. However, we may as well mention Peyton Rous, whose discovery of a viral cause of certain cancers was sidelined for decades (he received a Nobel prize when he was 87, 40 years after first being nominated https://en.wikipedia.org/wiki/Francis_Peyton_Rous!). Closer to (prokaryotic) home, Barry Marshall has edited a book (Marshall BJ (ed.): *Helicobacter* pioneers: firsthand accounts from the scientists who discovered helicobacters. Melbourne: Blackwell, 2002.) whose invited contributors had all long recognised a bacterial cause of ulcers and treated their patients accordingly, on the simple grounds that the antibiotics worked! Of course Marshall and Warren (and the wider world) knew nothing of this at the time of their discovery of *H. pylori*. Under these circumstances (as here) we rely on the overall weight of evidence (as much as its place of publication) to support our views. In Philosophy of Science circles this bolstering of a view via overlapping circles of self-consistent reasoning and data is referred to as ‘coherence’. Accordingly, in this sense, we have tried to make this a coherent story, and rehearse this point in the concluding section.
- *"In summary Kell, Potgieter and Pretorius have produced an interesting read which bring many important ideas to our attention. I am not convinced of the breadth of conditions to which they argue their ideas are applicable and I await with interest, demonstration of of how they may be practically pursued and some selected definitive proofs that iron-driven inflammatory disease is as important as they claim."*
  We have no further comments at this stage. Many thanks again for a very thoughtful review.
